# Species-specific partial gene duplication in *Arabidopsis thaliana* evolved novel phenotypic effects on morphological traits under strong positive selection

**DOI:** 10.1093/plcell/koab291

**Published:** 2021-12-07

**Authors:** Yuan Huang, Jiahui Chen, Chuan Dong, Dylan Sosa, Shengqian Xia, Yidan Ouyang, Chuanzhu Fan, Dezhu Li, Emily Mortola, Manyuan Long, Joy Bergelson

**Affiliations:** School of Life Sciences, Yunnan Normal University, Kunming, Yunnan, China; Department of Ecology and Evolution, The University of Chicago, Chicago, Illinois, USA; Department of Ecology and Evolution, The University of Chicago, Chicago, Illinois, USA; CAS Key Laboratory for Plant Diversity and Biogeography of East Asia, Kunming Institute of Botany, Chinese Academy of Sciences, Kunming, Yunnan, China; Department of Ecology and Evolution, The University of Chicago, Chicago, Illinois, USA; Department of Ecology and Evolution, The University of Chicago, Chicago, Illinois, USA; Department of Ecology and Evolution, The University of Chicago, Chicago, Illinois, USA; National Key Laboratory of Crop Genetic Improvement and National Centre of Plant Gene Research, Hubei Hongshan Laboratory, Huazhong Agricultural University, Wuhan, China; Department of Biological Sciences, Wayne State University, Detroit, Michigan, USA; CAS Key Laboratory for Plant Diversity and Biogeography of East Asia, Kunming Institute of Botany, Chinese Academy of Sciences, Kunming, Yunnan, China; Department of Ecology and Evolution, The University of Chicago, Chicago, Illinois, USA; Department of Ecology and Evolution, The University of Chicago, Chicago, Illinois, USA; Department of Ecology and Evolution, The University of Chicago, Chicago, Illinois, USA

## Abstract

Gene duplication is increasingly recognized as an important mechanism for the origination of new genes, as revealed by comparative genomic analysis. However, how new duplicate genes contribute to phenotypic evolution remains largely unknown, especially in plants. Here, we identified the new gene *EXOV*, derived from a partial gene duplication of its parental gene *EXOVL* in *Arabidopsis thaliana*. *EXOV* is a species-specific gene that originated within the last 3.5 million years and shows strong signals of positive selection. Unexpectedly, RNA-sequencing analyses revealed that, despite its young age, *EXOV* has acquired many novel direct and indirect interactions in which the parental gene does not engage. This observation is consistent with the high, selection-driven substitution rate of its encoded protein, in contrast to the slowly evolving EXOVL, suggesting an important role for EXOV in phenotypic evolution. We observed significant differentiation of morphological changes for all phenotypes assessed in genome-edited and T-DNA insertional single mutants and in double T-DNA insertion mutants in *EXOV* and *EXOVL*. We discovered a substantial divergence of phenotypic effects by principal component analyses, suggesting neofunctionalization of the new gene. These results reveal a young gene that plays critical roles in biological processes that underlie morphological evolution in *A. thaliana*.

## Teaser

A young gene produced by species-specific partial gene duplication quickly evolved critical developmental functions in Arabidopsis.

##  

**Figure:**
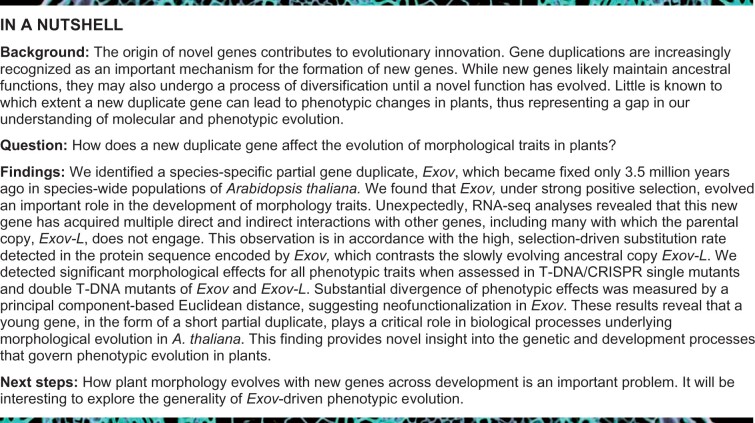


## Introduction

The origination of novel genes is an important process contributing to the evolution of organisms, as new genes have the potential to become genetic sources of evolutionary innovation (Chen et al., 2013; Long et al., 2013). Recent studies have identified lineage-specific and species-specific genes with important effects on diverse phenotypes, including development, sexual reproduction, brain functions, and behavior (Park et al., 2008; Chen et al., 2010; Ding et al., 2010, 2012; Zhang et al., 2011; Xia et al., 2016; Vankuren and Long, 2018; Lee et al., 2019). However, all of these studies have focused on metazoans, such as invertebrates, including fruit flies, and mammals. Little is known about the extent to which new gene evolution has coordinated phenotypic changes in plants, leading to a gap in our understanding of molecular and phenotypic evolution.

New genes typically arise through the duplication of existing genes at the DNA level, although a number of other mechanisms have been reported (Long et al., 2003; Zhang et al., 2019; Xia et al., 2021). These new genes may maintain functions similar to the parental gene or may undergo diversification until a completely novel function has evolved. Recently born genes, especially those appearing within the past few million years, provide excellent opportunities to study gene formation and associated phenotypic evolution, since all or most incipient changes are clearly recorded and preserved in extant organisms (Chen et al., 2013; Long et al., 2013). As such, one can relate evolutionary changes in genes to corresponding phenotypic expression.

In this study, we examined *EXOV* (At3g57110), a species-specific Arabidopsis (*Arabidopsis thaliana*) gene that originated in the Arabidopsis lineage 3.5 million years ago (MYA) through the duplication of the *EXOVL* (At5g60370) gene on chromosome 5, which was partially copied into a new locus on chromosome 3. We performed a comprehensive investigation of its phenotypic influence within an evolutionary context and analyzed the selective forces acting upon it. Our results revealed the unexpectedly large effects of this new gene on the evolution of morphological traits, demonstrating that new genes can drive rapid phenotypic evolution in planta.

## Results

### Evolutionary analysis of the new gene *EXOV* and its parental gene *EXOVL*

**Figure 1:**
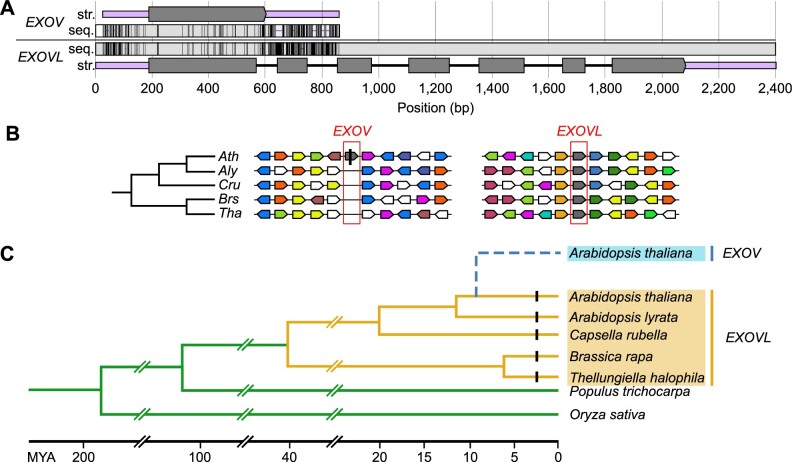
Evolution of *EXOV* (At3g57110), a partial gene duplication from *EXOVL* (At5g60370), as inferred from gene structure and syntenic analysis. A, Complete alignment of the sequence (seq.) and gene structure (str.) of the new gene *EXOV* (At3g57110) and the parental gene *EXOVL* (At5g60370). Gray boxes, exons; black line, introns; light purple box, untranslated regions (UTRs); black vertical lines in *EXOV* and *EXOVL* indicate unmatched nucleotides. B, Syntenic analysis of the new gene *EXOV* and the parental gene *EXOVL* based on the phylogenic tree. *Ath*, *Arabidopsis thaliana*; *Aly*, *Arabidopsis lyrata*; *Cru*, *Capsella rubella*; *Bra*, *Brassica rapa*; *Tha*, *Thellungiella halophila*. The red blocks highlight the orthologous regions of *EXOV* and *EXOVL* in the other four related species, showing no orthologous copies for *EXOV* and four orthologous copies for *Aly* (Aly496275), *Cru* (Carubv10026530m), *Bra* (Bra020254), and *Tha* (Thhalv10013696). Inspection of 10 genes that flank *EXOV* (the gray arrow block with vertical bar) and *EXOVL* (the gray arrow block) indicates orthologous syntenic arrangement of these genes in support of the orthologous comparison in the highlighted genomic regions of *EXOV* and *EXOVL* in the *A. thaliana* relatives. The arrows show the orientation of the genes. The colors represent homologous relationships and a color represents a distinct homologous gene. C, Phylogeny and divergence time between *A. thaliana* and its relatives and the species distribution of the new gene *EXOV* and the parental gene *EXOVL*.

We first describe the history of gene evolution in which the new gene *EXOV* was duplicated from the parental copy *EXOVL*, involving the movement from chromosome 5 to chromosome 3. The gene structure, alignment, sequences, and related molecular features are summarized in Figure 1, A, Supplemental Figure S1, and Supplemental File S1. Given the observed gene evolution, we explored the role of positive selection on the new gene.

#### A species-specific duplication between chromosome 5 and chromosome 3 gave rise to a new duplicate gene EXOV

Analysis of synteny indicated that the parental gene *EXOVL* has orthologs in all five related species investigated here: *A. thaliana*, sand cress (*Arabidopsis* *lyrata*), shepherd’s purse (*Capsella rubella*), field mustard (*Brassica rapa*), and salwater cress (*Thellungiella halophila*). Previous phylogenetic analyses estimated that *A. thaliana* split from *A. lyrata* ∼5 MYA (Beilstein et al., 2010), from *B. rapa* approximately 13–17 MYA (Town et al., 2006), and from *C. rubella* approximately 10–14 MYA (Koch and Kiefer, 2005). The new gene *EXOV* located on chromosome 3, which is a duplicate of a portion of the parental gene (Figure 1A) on chromosome 5, was present only in the *A. thaliana* genome (Figure 1, B and C)*.* This species-specific copy, *EXOV*, was detected in all *A. thaliana* accessions used in the population structural analyses of the 1001 Genomes Project (1001 Genomes Consortium, 2016), including the genomes of Columbia (Col-0) and Landsberg (La-0). These observations suggested that the new gene *Exov* is species-specific and has been fixed in *A. thaliana* since emerging after the recent split between *A. thaliana* and *A. lyrata*.

#### Detecting an asymmetrically high rate of substitution in EXOV in contrast to slow substitution in EXOVL

**Figure 2:**
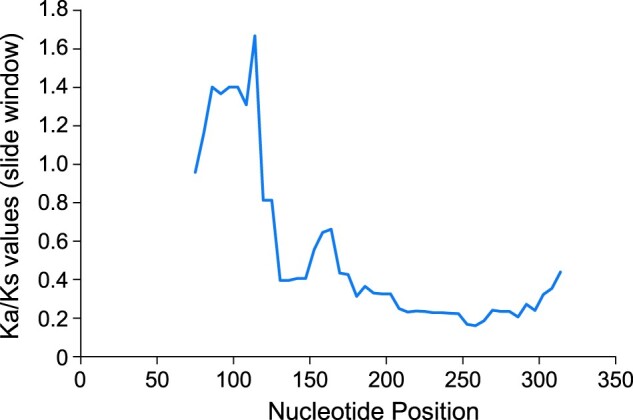
Ka/Ks sliding window analysis. Window length: 150 bp. Step size: 6 bp.

**Figure 3:**
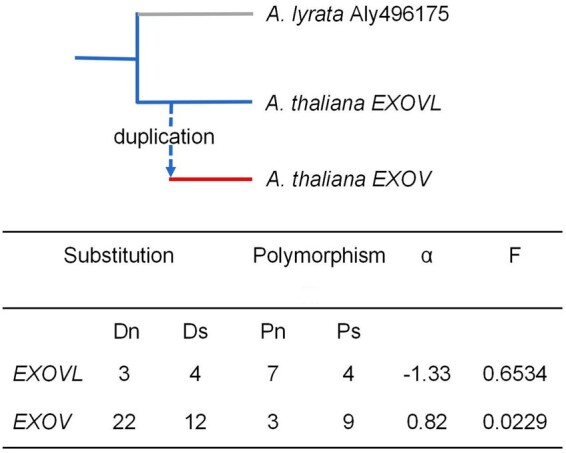
McDonald–Kreitman test of natural selection. The subscripts n and s indicate nonsynonymous and synonymous changes, respectively. α for *EXOV* is the proportion of substitution driven by positive selection; α for *EXOVL* may be the sampling error or segregation of deleterious mutations (Smith and Eyre-Walker, 2002). F, Fisher’s exact probability.

We performed a sliding window analysis of the   Ka (non-synonymous substitution rates)/Ks (synonymous substitution rates) ratio between *EXOV* and the duplicated portion of *EXOVL* within *A. thaliana*. The Ka/Ks ratio was higher than 1 in the first 100 bp, suggesting that this region is under positive selection. However, in the region between 120 and 400 bp, the Ka/Ks ratios between *EXOV* and *EXOVL* were <0.5 (Figure 2), together with an overall Ka/Ks 0.486 < 0.5 (Table 1), suggesting an evolutionary constraint on the protein-coding sequence in this region. Notably, the Ka value measuring divergence between *EXOV* and *EXOVL* was remarkably high for a duplicated region dating less than 5 million years (0.1063). Indeed, this rate was 3.01 times the Ka value (0.0353) between the *EXOVL* orthologs in *A. thaliana* and *A. lyrata*, which diverged earlier than the duplication time of *EXOV*. Taking *A. lyrata* and other more distant species, e.g. *C. rubella* and *B. rapa*, as outgroup species in a parsimony analysis, we detected an asymmetric distribution of substitutions accumulating in *EXOV* and *EXOVL* since the duplication event: 22 nonsynonymous substitutions in *EXOV* and only three nonsynonymous substitutions in *EXOVL* (Figure 3; “Materials and methods”); these values differed significantly from a null hypothesis of neutrality that predicts equal substitution between the two duplicates (2 = 14.44, *df *=* *1, *P *=* *0.0001).

**Table 1:** Ka/Ks ratio for the new gene *EXOV* and the parental gene *EXOVL*

Sequence 1	Sequence 2	Synonymous differences	Synonymous sites	Ks	Nonsynonymous differences	Nonsynonymous sites	Ka	Ka/Ks
*EXOV*	*EXOVL*	20.00	105.42	0.2187	30.00	302.58	0.1063	0.486
*EXOVL*	AL496175	37.50	282.50	0.1461	32.50	941.50	0.0353	0.242

The unexpectedly high rate of protein evolution in *Exov* implicated positive selection acting on *EXOV*. We took two approaches to test for putative positive selection: a population genetic test of selective sweeps; and an additional substitution analysis to compare with the population genetic prediction of neutrality (Nurminsky, 2001). However, before pursuing these approaches, it was necessary to understand the population structures of *A. thaliana* because demographic processes have the potential to affect the population genetic inferences and substitution analyses. Previous analyses detected significant population structures using then-large data sets in *A. thaliana*, revealing the need to consider demographic factors when testing selective forces (Nordborg et al., 2005; Horton et al., 2012). We used the much more expanded sequence information contained in the 1001 Genomes Project (1001 Genomes Consortium, 2016) to update previous population structure analyses for their incorporation into our population genetic analyses.

First, to infer population structure and assign accessions to populations, we used ADMIXTURE1.23 (Alexander et al., 2009), which adopts the likelihood model embedded in STRUCTURE (Raj et al., 2014). To cluster all accessions on the basis of geographic distribution (Supplemental Data Set S1), we analyzed the data by successively increasing *K* from 2 to 8 (Supplemental Figure S2) using the ADMIXTURE likelihood algorithm. The cross-validation error was smallest when *K* = 8 (Supplemental Figure S3A), revealing clear global population structure among these eight subgroups (Supplemental Figure S3B). The population structure was consistent with earlier analyses (Nordborg et al., 2005; Horton et al., 2012) that detected population clustering, but with most polymorphisms shared species-wide.

This, and previous observations of global population structure across the *A. thaliana* genome (Nordborg et al., 2005; Wright and Gaut, 2005), revealed potential demographic processes that render tests of positive selection too liberal if a comparison is made to a theoretical distribution, which may cause a deviation from expected values for the Tajima’s D test, the Fay–Wu test, the Fu–Li tests (Tajima, 1989; Fu and Li, 1993; Fay and Wu, 2000), even in the absence of positive selection. We therefore computed the empirical distributions of these statistic tests across the entire genome (Supplemental Data Set S2 and Supplemental Figure S4) using the worldwide accessions (from the 1001 Genomes Project, Supplemental Data Set S1). Compared to these empirical distributions, we failed to find significance for any of the above population genetic statistics calculated for the *EXOV* and *EXOVL* genes (Supplemental Figure S4), suggesting that neither *EVOV* nor *EVOVL* has undergone a selective sweep.

We next used the McDonald–Kreitman test (McDonald and Kreitman, 1991) to test for positive selection on the substitutions of *EXOV* (Supplemental Table S1). This test detects the proportion of amino acid changes occurring between species and compares this to the proportion occurring within a species, considering the evolution of a protein-coding gene in two closely related species. In this test, we compared the polymorphisms within *EXOV* in *A. thaliana* to sequence divergence between *EXOV* in *A. thaliana* and the two outgroup species, *A. lyrata* and *C. rubella.* We also performed the same test for *EXOVL*, comparing polymorphisms within species to divergence between species.

We furthermore assigned divergence between *EXOV* and *EXOVL* to each lineage since the duplication event and measured the time since the duplication by counting the number of shared synonymous substitutions in *EXOV* and *EXOVL* that occurred between the speciation of *A. thaliana* and the duplication of *EXOV*. Two of six *EXOVL-*specific synonymous substitutions were shared with *EXOV* (those at sites 204 and 216), suggesting that *EXOV* was duplicated soon after the speciation of *A. thaliana.* We estimated that the duplication occurred 3.5 MYA, roughly one-third of the time since emergence of the *Arabidopsis thaliana* 11 MYA (Yogeeswaran et al., 2005).

For the McDonald–Kreitman test, we counted polymorphisms in synonymous and nonsynonymous sites in *EXOV* and the duplicated portion of *EVOVL* in a data set of 709 *EXOV* sequences and 455 *EXOVL* sequences computationally extracted from the *A. thaliana* accessions in the 1001 Genomes Project (1001 Genomes Consortium, 2016; Figure 3;  Supplemental Table S1). In only 3.5 million years, EXOV changed its sequence dramatically: 22 nonsynonymous substitutions led to a modification of 21 (or 15%) of the 136 amino acid residues that this gene encodes (Figure 3). In contrast, the ancestral region of *EXOVL* evolved slowly, with only three amino acid changes. The McDonald–Kreitman test detected strong positive selection acting on *EXOV* (Fisher’s exact test: two-tailed *P *=* *0.0229). A high value (=1–Neutral Index) of 0.82 revealed that most of the detected amino acid substitutions on *EXOV* were driven by positive selection. *EXOVL*, however, evolved slowly, showing no signal of positive selection except, perhaps, a segregation of deleterious genetic variation, as its negative value (−1.33) suggested.

### Molecular and expression analyses of *EXOV* and *EXOVL*

Given that our evolutionary analysis revealed a signature consistent with a functional gene evolving under natural selection, we sought signals of functional evolution. First, we investigated changes in the molecular structure and sequence that have the potential to underlie functional change. Second, we assessed differences in the expression patterns of new and parental genes.

#### The new gene EXOV is a duplication of the 5ʹ-end of the parental gene EXOVL

To understand the functional significance of the new gene *EXOV*, we investigated the relationship between evolutionary changes in *EXOV* and known molecular functions of the parental gene *EXOVL*.

We first examined the evolution of the parental gene *EXOVL*. Sequence alignment of EXOVL and its orthologs revealed high conservation from mammalian to plant species, especially within the N-terminal region in plants (Supplemental Figure S5A). Sequence alignment of EXOVL and its orthologs also showed high similarity along the C-termini of the proteins, which bear the exonuclease domain as in yeast (*Saccharomyces cerevisiae*) Exo5p and human EXO5 (Yeeles et al., 2009; Burgers et al., 2010; Sparks et al., 2012). One unique feature of this catalytic domain is its iron-sulfur cluster structure motif, which is an essential component of many DNA and RNA processing enzymes (White and Dillingham, 2012). The cysteine residues that form the critical Fe–S cluster motif in EXOVL and its orthologs in mammals and zebrafish (*Danio rerio*) were identical (Supplemental Figure S5A).

As shown in Figure 1A, the new gene *EXOV* was a partial duplicate from the 5′-end of the parental gene *EXOVL*, corresponding to exon 1 (which encodes the Exo5p homologous catalytic domain). Although EXOVL in plants was highly conserved in the N-terminal region, especially at positions R63, K85, and D103 (Supplemental Figure S5B), the conserved polar charged residues in the parental protein have been replaced in EXOV with the more neutral histidine, isoleucine, and tyrosine residues, respectively (Supplemental Figure S5). The corresponding region of the ATP-dependent helicase/deoxyribonuclease subunit B AddB regulates catalytic activity by forming contacts with AddA subunits (Supplemental Figure S5C). In contrast to the conservation defined by the parental protein EXOVL, which may be involved in fine-tuning catalytic activity during DNA metabolism (Burgers et al., 2010; Sparks et al., 2012), the N-terminal region of the new protein EXOV has accumulated many changes, indicating that EXOV has evolved a smaller and distinct protein sequence with a diverged function.

#### Expression profiles of the new gene *EXOV* and the parental gene *EXOVL* are overlapping

**Figure 4:**
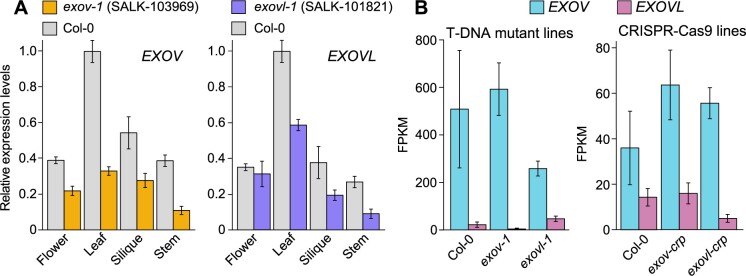
Expression analyses of mutants for *EXOV* and *EVOVL* using RT-PCR and RNA-seq*.* A, Relative *EXOV* and *EXOVL* expression levels in flowers, leaves, siliques, and stems from the WT Col-0 and the T-DNA insertion mutants *exov-x* (left) and *exovl-1* (right). Relative expression levels in Col-0 leaves were set to 1. Data are shown as mean ± se of triplicate experiments. The *t* tests for comparison between WT and *exov* are significant, except in silique: *exov*: flower, *P* = 0.0015; leaf, *P* = 0.0015; silique, *P* = 0.3861; stem, 8.82e−05. *exovl*: flower, *P* = 0.0101; leaf, *P* = 0.0408; silique, *P* = 0.0031; stem, *P* = 0.0069. B, Expression levels of *EXOV* and *EXOVL*, as determined by RNA-seq of whole plants, presenting as FPKM. *exov-1* and *exovl-1* are T-DNA insertions; *exov-crp* and *exovl-crp* are CRISPR/Cas9 alleles. WT is Col-0. Data are shown as mean ± se for three biological replicates. *t* Tests for *exovl* versus WT, *P* = 0.0168; for *exov* versus WT, *P* = 0.1230. *t* Tests for CRISPR mutants: *exov-crp* versus WT, *P* = 0.7957; *exovl-crp* versus WT, *P* = 0.3524.

To quantify the expression of new and parental genes, we performed reverse transcription quantitative polymerase chain reaction (RT-qPCR), using various tissues collected from wild-type (WT) T-DNA insertion mutant plants. *EXOV* and *EXOVL* were both transcribed in all tested organs: leaves, stems, flowers, and siliques (Figure 4A). We also compared the expression levels of the two genes in the WT using the eFP database (Klepikova et al., 2016). The new gene *EXOV* displayed a low expression level in specific tissues, such as leaves and siliques, while the parental gene *EXOVL* showed relative high expression level in leaves and siliques. Both new gene and parental gene were highly expressed in flowers (Supplemental Figure S6). The results of our RT-qPCR experiments revealed that when compared to the WT, the T-DNA insertion mutants *exov* and *exovl* exhibited much lower expression of their respective genes in all tissues tested, where the expression was often reduced by as much as ≥50% (Figure 4A).

As a complement to RT-qPCR and in silico analyses, we conducted a transcriptome deep RNA sequencing (RNA-seq) analysis from 6- to 8-week-old WT and T-DNA insertion mutant plants. As *EXOV* was duplicated from *EXOVL*, their sequences were mostly identical (Figure 1A;  Supplemental Figure S5B). Because we could not distinguish the source of reads that mapped to both genes, we opted to report only reads uniquely mapping to *EXOV* or *EXOVL* in each sample. The T-DNA mutants of *EXOV* and *EXOVL* showed significant or marginally significant reduction in expression, by as much as 50% (Figure 4B, T-DNA insertion lines). We also compared the transcriptome of the T-DNA mutants *exov* and *exovl* to that of the WT, which revealed changes in the expression of 819 genes, of which 255 were shared between the two mutants. Another 361 differentially expressed genes were unique to the *exov* mutant, with the remaining 203 genes being specifically differentially expressed in the *exovl* mutant. These data provided evidence for a functional divergence after the duplication of *EXOV* from *EXOVL*, suggesting that *EXOV* and *EXOVL* each carry out shared but also unique functions (Supplemental Table S2).

#### The new gene EXOV evolved to regulate additional biological processes beyond those regulated by the parental gene EXOVL

**Figure 5:**
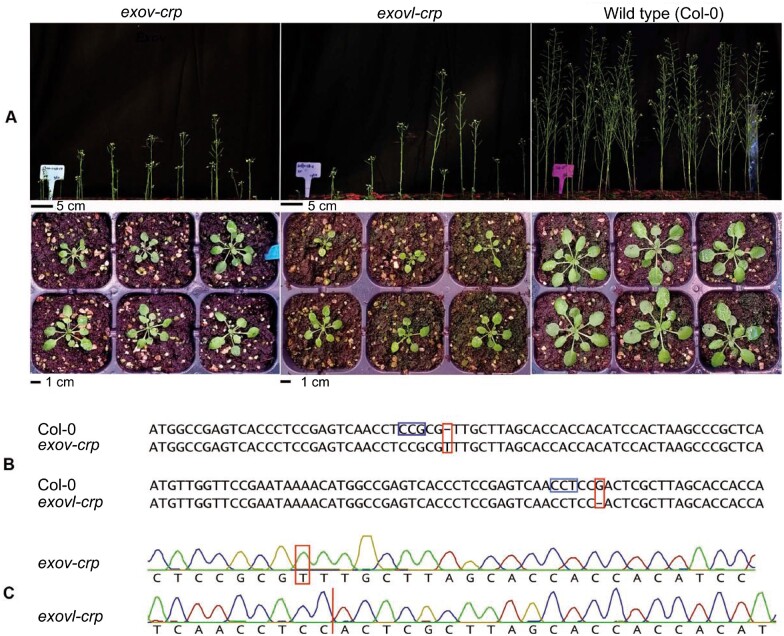
Generation of CRISPR/Cas9 mutants and measurement of their phenotypic effects. A, Phenotypes of T_3_ transgenic plants of the sgRNA target. *EXOVL* (At5g60370) and *EXOV* (At3g57110) CRISPR-Cas9 allele: *exov-crp* and *exovl-crp* T_3_ transgenic lines exhibited a small-seedling phenotype compared to the WT Col-0. Similar to the T-DNA mutants *exov* and *exovl*, they showed dwarfed and retarded growth. B, Genomic sequence alignment between Col-0 and *exov-crp* or *exovl-crp*. Representative sequences of mutant alleles of *exov-crp* and *exovl-crp* T_2_ transgenic lines. The WT sequence is shown on top as reference with the PAM highlighted in the red frame. Nucleotide deletion and insertion in the mutants are highlighted in the blue frames. C, Representative electropherograms showing gene editing at the target regions.

To better understand how the species-specific *EXOV* gene diverged in its function as a consequence of distinct mutations, we generated specific mutations in *EXOV* and *EXOVL* (*exov-crp* and *exovl-crp* alleles) using the clustered regularly interspaced short palindromic repeats (CRISPRs) and CRISPR-associated protein 9 nuclease (Cas9) system (Supplemental Figure S1). We identified the genome-edited mutants *exov-crp* harboring a 1-bp insertion and *exovl-crp* with a 1-bp deletion (Figure 5). To assess changes in expression levels, we performed an RNA-seq analysis of the WT, *exov-crp*, and *exovl-crp*; (Figure 4B). In contrast to the T-DNA mutants, the expression levels of *EXOV* and *EXOVL* in the *exov-crp* and *exovl-crp* mutants did not appear to change significantly relative to the WT, again focusing only on reads mapping uniquely to each gene (for all *t* tests, 0.7957 > *P *>* *0.1208; Figure 4B). We suspected that the specific single-nucleotide changes in *exov-crp* and *exovl-crp* do not target the regulatory regions. In addition, the functional consequences of the insertion (*exov-crp*) and deletion (*exovl-crp*) would affect the open reading frame by introducing a frameshift (Figure 5, B and C). The asymmetric correlation in expression levels for the parental gene and new gene mutants and WT support a functional divergence after the duplication of *EXOV* from *EXOVL*.

Based on the transcriptome data, we identified differentially expressed genes in each mutant compared to the WT. Specifically, 967 genes were downregulated, and 153 genes were upregulated in *exov-crp* relative to the WT, while 750 genes were downregulated and 198 genes were upregulated in *exovl-crp* (Supplemental Data Set S3). Surprisingly, the new gene appeared to interact with more genes (1,120 genes being down regulated or upregulated if mutated, including both direct and indirect interactions) than did the parental gene (948 being downregulated or upregulated if mutated; *X^2^*=18.511, *P = *1.689×10^−5^, under the null hypothesis of equal number of interacting genes). We observed the same pattern in T-DNA-insertion mutants, with 616 genes being downregulated/upregulated (535/81) in *exov* compared to 458 genes being downregulated/upregulated (340/118) in *exovl* (*X*^2^*=*26.863, *P = *2.185×10^−7^) (Supplemental Data Set S4), thus providing a striking example of a recently formed gene evolving more interactions with other genes in the genome than the parental gene. This observation contrasts with the conventional view that new genes are integrated into the ancestral gene–gene interaction network and remain less integrated into cellular networks than old genes. It also provides a counter example to the observation of reduced levels of co-expression for new genes in mammalian evolution (Zhang et al., 2015).

We ranked the differentially expressed genes based on the *P*-values for simple *t* tests comparing the WT and CRISPR/Cas9 mutants and used this ranked list as input for gene ontology (GO) annotation via the online tool Gorilla with default running parameters (Supplemental Figure S7). The results highlighted a unique set of enriched GO terms that were identified at different cutoffs, including pollen tube development, pollination, multicellular organism processes, cell tip growth, cell morphogenesis involved in differentiation, developmental cell growth, pollen tube growth, aging, movement of the cell or subcellular components, and actin filament-based movement. While both the parental and new genes may be involved in aging, the new gene appeared to additionally regulate additional biological processes such as the movement of the cell or subcellular components, including actin filament-based movement (Supplemental Figure S7), potentially explaining its increased genetic interactions. The information from the GO analyses suggested a valuable, albeit broad, picture of genetic mechanisms that, with further analysis, would enhance our understanding of the evolutionary forces on the parental and new genes that we investigated.

### Detection of the phenotypic effects of *EXOV* and *EXOVL* on morphological traits

Our evolutionary analyses detected signatures of positive selection in the gene sequences, as well as the evolution of hundreds of new expression interactions involving the new gene. These evolutionary changes at the sequence and transcriptome levels would be expected to have functional repercussions. To understand the functional divergence of *EXOV* and *EXOVL*, we scored seven important developmental traits in both WT plants and mutants.

#### Seven morphological traits exhibit significant phenotypic effects in *exov* and *exovl* mutants

**Figure 6:**
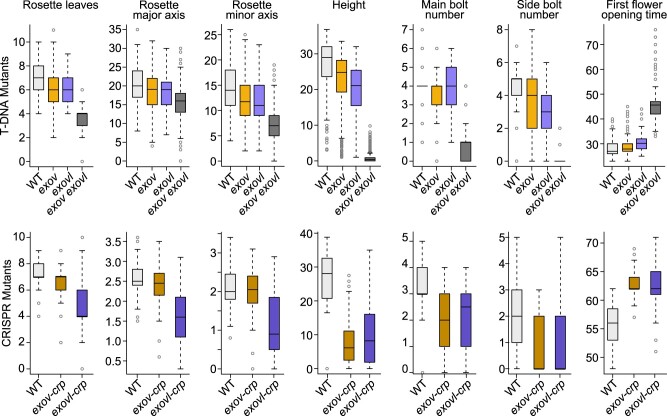
Distribution of phenotypic effects on seven traits in the *exov, exovl* single mutants and the *exov exovl* double mutant. Top: T-DNA insertions; Bottom: CRISPR knockouts. WT, wild type (Col-0). Data are shown as boxplots, with the horizontal line indicating the median; the box indicates the 25th and 75th percentiles.

We measured six growth traits (number of rosette leaves, main and side bolt number, height, rosette major, and minor axis) and the flowering time of WT plants, as well as the single T-DNA insertions and CRISPR/Cas9 knockout mutants of *EXOV* and *EXOVL* and the double mutant *exov exovl* (Supplemental Figure S8 and Supplemental Table S3). In general, the *exov* and *exovl* mutants showed significant phenotypic effects compared to the WT in all seven traits examined (Figure 6;  Supplemental Table S3). In 21 comparisons between T-DNA insertions (*exov, exovl*, and *exov exovl*) and WT, all were significant with *P *≤* *0.00001 except *exovl* for branch number on the main bolt, which was not significantly different from the WT (Wilcoxon rank sum test; a Gaussian-based test gave similar results). Among 14 comparisons between the CRISPR knockouts (*exov-crp and exovl-crp*) and the WT, all were significant with *P *≤* *0.00001 (Supplemental Table S3). We observed that *exov* and *exov-crp* plants are petite and display reduced growth rates (Figure 5A). Remarkably, these mutants of the new gene *EXOV* frequently showed phenotypic effects as strong as mutants in the parental gene *EXOVL*, whereas three traits showed an even stronger effect in *exov* mutants than in *exovl* mutants (*exov* for number of leaves; *exov-crp for* height; *exovl-crp for* main number of bolts; Figure 6; Supplemental Table S3). In general, we observed that all morphological traits examined differ significantly between the WT and mutants of the new gene and parental gene. Additionally, compared to the WT, the *exov-crp* and *exovl-crp* mutants were petite and displayed reduced growth rates. In general, the *exov-crp* allele showed phenotypic effects similar to those of the *exovl-crp* allele, while two traits, height and main bolts number, showed a stronger phenotypic effect in *exov-crp* than in *exovl-crp*.

Furthermore, *exov exovl* double mutant plants showed a strong and significant change in all seven traits tested relative to the single mutants and the WT (*P *<* *2×10^−16^, Wilcoxon rank sum test; the Gaussian-based test gave similar results; (Figure 6, top; Supplemental Table S3). This observation suggested that the genetic bases of phenotypic changes in the two genes do not completely overlap. For example, while the height of the main bolt reached 20–30 cm in 40-day-old plants with the four single mutants tested and the WT, the double mutant did not produce a bolt within the same time frame. In addition, the first flower opened at least 15 days later in the double mutant relative to the single mutants and the WT, suggesting stronger effects of the double mutant on these seven morphological traits.

We will note here that we determined the insertion sites for all T-DNA insertion mutants, including the three alleles for the new gene, using whole-genome sequencing. We detected no additional insertion sites in the mutant genomes. Also using whole-genome sequencing, we confirmed that the CRISPR knockout lines are specific knockouts for their intended target gene, with no obvious off-targets in other parts of their genome.

#### Principal component analyses detected segregation of the phenotypic effects of mutants for EXOV and EXOVL from *WT* genes

**Figure 7:**
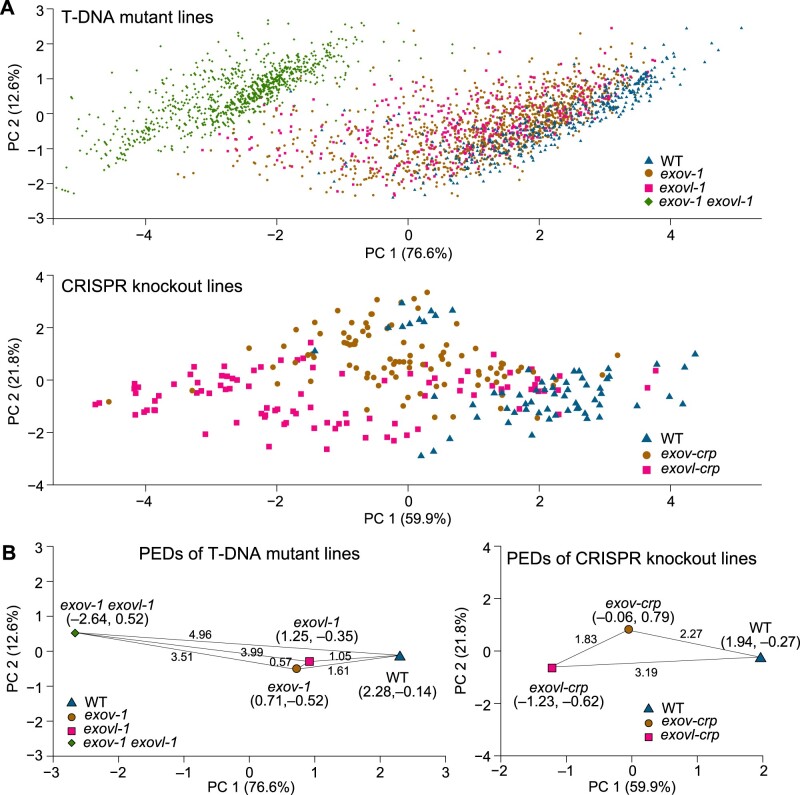
PCA of the phenotypic effect of the new and parental genes and their PED distances. A, Distribution of principle components of mutant lines. Number of individuals for each genotype: *exov-1*, 1,098; *exovl-1*, 389; double mutant, *exov-1 exovl-1*, 1,028; WT (Col-0), 413; *exov-crp*, 96; *exovl-crp*, 96; WT, 64. B, Distances of phenotypic evolution among mutants. The PED distance among mutants, defined as a geometric distance using the average values of PC1 and PC2 for each population (the pairs of coordinates in PC1 and PC2 respectively are given under each mutants and WT).

We employed principal component analysis (PCA) to obtain a global view of the differences between the phenotypes and across the mutants, as represented in the assembled data and described in Figure 6 and Supplemental Figure S9. PCA components 1 and 2 contributed 58.8% and 14.5% for T-DNA insertion and 59.9% and 21.8% for the CRISPR mutants, respectively, of the total eigenvalues (Figure 7A).

Interestingly, the two components in the two types of mutants showed remarkable segregation among WT, new gene mutant, and parental gene mutant plants, as shown in Figure 7B. First, it was evident that mutants of both the new and old gene cause shifts away from the WT, underscoring the strong effects of these mutants on the overall phenotypes. Second, the *exov* and *exovl* single mutants exhibited distinct and separate distributions, revealing that the phenotypic effects associated with the loss of EXOV differ from that of EXOVL. Third, the long distances, 3.99 and 2.20, of phenotypic evolution (PED) of the *exov exovl* double mutant from their *exov* and *exovl* constituent single mutants illustrated additional phenotypic effects that are larger than the effects of the single mutants, with PED distances of 1.05 and 1.62, respectively, relative to the WT. These numbers reflected strong epistasic effects evolved by both *EXOV* and *EXOVL*. Finally, the T-DNA insertion mutants and CRISPR knockouts showed a difference in PED values between the single mutants and the WT; for the T-DNA insertions, the PED of *exov* was greater than that of *exovl*, whereas the CRISPR knockouts followed an opposite relationship with *exov* < *exovl*. This difference may reflect the different functional consequences of the mutations at the transcriptional and translational levels. Overall, the clear segregation of *exov* mutants (*exov* and *exov-crp*, blue) away from the WT and the mutants of the parental gene *EXOVL* reveals that the species-specific gene *EXOV* evolved novel and strong phenotypic effects in a period of time as short as 3.5 MYA.

## Discussion

As our ability to study the roles of new genes in phenotypic evolution has become feasible, the importance of these genes is becoming apparent. Two studies in *A.* *thaliana* have demonstrated that three recently evolved duplicate genes from the cytochrome P450 family (*CYP98A9*, *CYP98A8*, and *CYP84A4*) assembled two new biochemical pathways related to phenolic metabolism required for pollen development and α-pyrone biosynthesis (Matsuno et al., 2009; Weng et al., 2012). How plant morphology evolves and what the genetic basis underlying morphological evolution is of central importance in the field of new gene research. Here, we detected that a species-specific duplicated gene has rapidly evolved a series of species-specific phenotypic effects that have affected morphological traits in *A.* *thaliana*. This was not predicted by conventional theories of phenotypic evolution and gene evolution. As important as the former two study examples, the present study reveals that a species-specific gene plays an important role in the phenotypic evolution of *A. thaliana*.

Our nucleotide substitution analyses revealed a Ka/Ks ratio much less than 1 in the new gene, *EXOV*, suggestive of its strong selective constraints (Table 1 and Figure 2). Despite the young age of *EXOV*, which was generated through gene duplication ∼3.5 MYA, its divergence in nonsynonymous sites from *EXOVL* reached a surprisingly high level of 14%. Furthermore, the McDonald–Kreitman test detected a significant excess of nonsynonymous substitutions compared to the within-species variation at nonsynonymous and synonymous sites. These analyses further determined that the protein encoded by *EXOV* evolved approximately seven times more rapidly than that of *EXOVL*, suggesting the significant impact of positive selection driving the neofunctionalization of *EXOV*.

The protein encoded by the old gene, *EXOVL*, possesses a highly conserved domain associated with exonuclease functions in the yeast ortholog Exo5p (previously named Dem1p for defects in morphology; Burgers et al., 2010; Sparks et al., 2012)*.* However, we identified no conserved domains in the protein encoded by the new gene *EXOV*, suggesting that the recent appearance in *A. thaliana* of this novel gene may lead to a new function. Both new and old proteins are predicted to localize to the chloroplast, based on chloroplast transit signal predictions (Bosco, 2003). The homologous genes to *EXOVL* are highly conserved across humans and yeast, where they has been shown to be involved in DNA metabolism and genome stability of mitochondria (Burgers et al., 2010; Sparks et al., 2012).

Our prediction that the new gene *EXOV* is functional is further supported by the significant phenotypic effects on the morphological traits in T-DNA and CRISPR/Cas9 mutant lines. Interestingly, the new gene *EXOV* showed a robust signal indicating positive selection in the 5′-end of the gene. The residues of the corresponding regulatory domain evolved to give rise to new functional roles to EXOV, but the putative catalytic domain was lost. This type of protein evolution implicates a fundamental role for proteins to gain new functions.

Furthermore, we found significant segregation of the phenotypic effects of the new gene versus the old gene among seven at least partially independent traits. We detected strong evidence for functional divergence introduced by the new gene by PCA. The distribution of PCA scores showed functional shifts between mutants for the new gene and old gene. Unexpectedly, given the young age of *EXOV*, these analyses detected a tremendous divergence from the parental gene to this new, species-specific gene, suggesting its critical roles in the evolution of morphological traits. Surprisingly, the T-DNA insertions and CRISPR knockouts revealed that the new gene *EXOV* can have equal or stronger effects than the old parental duplicate copy *EXOVL* on a few morphological straits. Whole-genome sequencing of the mutant lines confirmed that these phenotypic effects are not caused by background mutations such as additional T-DNA insertions or off-target genome editing elsewhere in the genome. Furthermore, that the multiple mutant lines revealed similar phenotypic effects support the notion that the observed phenotypic effects are consequence of the mutations created in these lines.

Our data on *EXOV* show that a short gene duplicate quickly evolved critical developmental function. The current genomic era is generally confronted with numerous short gene fragments in genomic annotation. For example, many intact transposable elements in maize (*Zea mays*; Jiao et al., 2017) contribute to the relatively short duplicated gene fragments (Schnable et al., 2009). Genomic annotations often ignore these genetic elements, which are instead treated as useless sequence noise or pseudogenes. This practice in genomic annotation would cause an underestimate of the number of genes responsible for important phenotypic functions. Our study presented a cautionary note for current genomic annotations, which we believe need improvement to better handle short gene fragments.

Although both new and parental genes may be involved in the biosynthesis of secondary metabolites, the RNA-seq comparison between the mutants and WT revealed that the new gene evolved many more genetic interactions than the old gene (Supplemental Table S4). The large number of interactions suggests a hub in genome interaction networks, potentially explaining its significant impact on morphological trait divergence and the strong epistasis effects detected in T-DNA double mutants (Figure 7). These newly evolved interactions give insight into evidence for positive selection on phenotypic evolution, as well as suggesting that the new gene may have contributed to the phenotypic evolution underlying the examined morphological traits in *A. thaliana* through neofunctionalization.

## Materials and methods

### Plant materials and growth conditions

Arabidopsis seeds were surface sterilized with 50% (v/v) commercial bleach for 5 min and then rinsed five times with sterile water. Following stratification for 2–3 days at 4°C, Arabidopsis seeds from the related species *A. thaliana*, *A. lyrata* subsp. *lyrata*, *A. lyrata* subsp*. petraea*, and *A. halleri* were released under a long-day photoperiod (16-h light/8-h dark) at 22°C in the University of Chicago greenhouse and grown for 5–6 weeks. The soil was a mix of 50% Berger BM-1 and Berger BM-2 professional growing media (Berger Peat Moss LTD; www.berger.ca). Illumination was provided by Philips Ceramalux High Pressure Sodium lamps (430 W).

The Arabidopsis T-DNA insertion lines for *EXOV*, *exov-1* (SALK_103969), *exov-2* (SALK_036494), *exov-3* (SALK_064431), and for *EXOVL*, *exovl-1* (SALK_101821) were ordered from the Arabidopsis Biological Resource center at Ohio State University (http://www.arabidopsis.org/). The locations of the T-DNA insertions were confirmed by PCR using the T-DNA border primer LBb1.3 and gene-specific primer pairs for both new gene and parental gene. Plants with a homozygous T-DNA insertion were identified by screening self-fertilized progeny from the mutants using PCR amplification. Homozygous lines were identified by negative LP-RP amplification and positive LBb1.3-RP amplification. The exact DNA insertion positions were verified by sequencing the LBb1.3-RP PCR products. Primers for genotyping are listed in Supplemental Table S5. Double mutant lines were obtained by crossing SALK_101821 with SALK_103969, SALK_036494, or SALK_064431, respectively. Homozygous *exov exovl* double mutant plants were identified by using 4xPCR reactions, showing negative LP-RP amplification and positive LBb1-RP amplification of both genotypes. Homozygous plants for T-DNA insertion lines were used to evaluate phenotypic changes compared to the WT accession Columbia-0 (Col-0). For phenotypic assessment of the double mutant, *exov-1 evovl-1* was used. The consistent phenotypic effects among the T-DNA lines for single and double mutants and the knockout lines created by CRISPR/Cas9 further suggest that both T-DNA and CRISPR/Cas9 lines are lacking substantial background mutations, including additional insertions of the T-DNA.

### Generation of the *exov-crp* and *exovl-crp* mutants using CRISPR/Cas9

A modified version of vector pCAMBIA1300 (pCAMBIA1300-YAO-Cas9) harboring a *Cas9* expression cassette was used to introduce mutations in *EXOV* and *EXOVL* (Yan et al., 2015). The CRISPR/Cas9 constructs were transformed into *A. thaliana* WT Col-0 through floral dipping (Clough and Bent, 1998). Primary transformants were selected either based on red fluorescence or for resistance to 16 mg L^−1^ hygromycin on full-strength Murashige and Skoog medium with 3% (w/v) sucrose. Genomic DNA extracted from leaf tissues of 2-week-old hygromycin-sensitive T_2_ seedlings was used as template for PCR. To screen induced mutations at the *EXOV* and *EXOVL* targets, amplicons were generated that overlap the sgRNA target sites with gene-specific primers (Supplemental Table S5) from Cas9-free plants. Homozygous T_2_ transgenic lines (*exov-crp, exovl-crp*) were identified by sequencing the above PCR products and by whole-genome sequencing, as below.

### Identification of mutation sites of T-DNA lines and CRISPR lines

To identify mutation sites, sequencing libraries for the genomes of T-DNA mutant lines *exov-1* (lines SALK-103969-4 and SALK-103969-60), and *exovl-1*, CRISPR mutant lines *exov-crp-4* (lines 57110-4 and 57110-1-15), and *exovl crp* (line 60370-4) were prepared using the TruePrep DNA Library Prep Kit V2 for Illumina (Vazyme #TD501) and sequenced on an Illumina HiSeq X Ten platform. Whole-genome sequencing data were generated with a genome coverage >99% and a read depth of at least 50 (Supplemental Table S6).

For T-DNA insertion mutants, raw reads were de novo assembled by SOAPdenovo2 (Luo et al., 2015) and chimeric sequences bridging the T-DNA plasmid and the Arabidopsis genome were identified by BLAST-like alignment tool (Kent, 2002). For CRISPR mutants, raw reads were first mapped to the TAIR10 reference genome (Berardini et al., 2015) by BWA (Li and Durbin, 2010) and VCF files were generated by GATK (Van der Auwera et al., 2013) and corrected with 1,001 genomes (1001 Genomes Consortium, 2016). On-target and off-target sites were then predicted by the online tool CRISPR-P 2.0 (Liu et al., 2017); mutation sites were retrieved in 100-bp regions centering on the expected target loci. Furthermore, mapping T-DNA insertion sites were conducted by fusion primers and nested integrated PCR (FPNI-PCR; Wang et al., 2011). The potential on-target and off-target sites were mapped to the genome sequence. Target products through FPNI-PCR including T-DNA insertion flanking sequence and target genome sequence were sequenced and mapped to the *A. thaliana* genome by BLAST to confirm the insertion positions.

The single T-DNA insertion sites were identified based on whole-genome sequencing data (Supplemental Table S6 and Supplemental Data File S2). Outside of the target positions, no insertion was mapped to other positions along the chromosomes. The flanking sequences indicated that the T-DNA insertion in the *exov-1* allele is between 21,134,854 and 21,135,628 bp on chromosome 3 and that for *exovl-1* is between 24,283,931 and 24,291,840 bp on chromosome 5 (Supplemental Table S6).

For CRISPR lines, we used the entire genomes from the 1,135 accessions of the 1001 Genomes Project as background to filter off-target sites. No off-targets were detected in either *exov* or *exovl* CRISPR lines. Genome editing was confirmed in the *exov-crp* line (1-bp insertion) and in the *exovl-crp* line (1-bp deletion; Supplemental Tables S6 and S7).

### Targeted DNA sequencing

The new gene *EXOV* and the parental gene *EXOVL* were PCR amplified from genomic DNA in four separate reactions using the primer pairs in Supplemental Table S5. Following PCR, the amplified products were sequenced from both strands using each gene-specific primer, BidDye chemistry, and a 3730 automated sequencer (Applied Biosystems).

### RT-qPCR

Total RNA was extracted from leaves, flowers, young siliques, and stems collected from the WT and mutants using the Eastep Super Total RNA Extraction Kit (Promega) and reverse transcribed using the Reverse Transcription System (Promega) according to the manufacturer’s protocol. RT-qPCR was performed with the ABI7500 real-time PCR system using TransStart Top Green qPCR SuperMix (TransGen, Beijing, China). Relative gene expression levels were calculated by normalizing against the internal control *ACTIN 8*. Three biological replicates were carried out for each sample. All primers used for RT-qPCR are listed in Supplemental Table S5.

### RNA-seq analysis

Entire plants from the WT and relevant mutants growing under a long-day photoperiod (16-h light/8-h dark at 22°C) in the Kunming Institute of Botany (KIB) greenhouse for 6–8 weeks were collected and frozen in liquid nitrogen for RNA extraction and sequencing, including leaves, flowers, stems, and all other tissues. Total RNA was extracted with Trizol reagent from three biological replicates of WT *A. thaliana*, the T-DNA mutants (*exov* SALK-103969 and *exovl* SALK-101821), and CRISPR/Cas9 mutants (*exov-crp, exovl-crp*). mRNAs were purified using an Oligotex mRNA Mini Kit (QIAGEN). Next, cDNA libraries were prepared using the mRNA-seq Sample Preparation Kit (Illumina) following the nonstrand-specific protocol. Briefly, mRNAs were fragmented by exposure to divalent cations at 94°C, and fragmented mRNAs were converted into double-stranded cDNA. Then, cDNA ends were polished with 39-hydroxyls extended with A bases and ligated to Illumina-specific adapter-primers. The resulting DNA was amplified by 15 cycles of PCR followed by purification using the Qiagen PCR Purification Kit to obtain the final library for sequencing on an Illumina HiSeq2000 platform as 100-bp paired-end reads. The DNA yield and fragment insert size distribution of sequencing libraries were determined on an Agilent Bioanalyzer. A summary of read numbers per genotype and replicate is given in Supplemental Table S9. Tophat version 2.0.12 was used to map reads to the *A. thaliana* TAIR10 reference genome. Next, cuffdiff version 2.2.1 was used to find differentially expressed genes between samples (Trapnell et al., 2012), which were then applied to GOrilla for GO enrichment analysis (Eden et al., 2009). When looking at *EXOV* and *EXOVL* expression in the mutants, we counted uniquely mapping reads using HTSeq with “union” mode (Anders et al., 2014).

### Measurement of phenotypes

**Figure 8:**
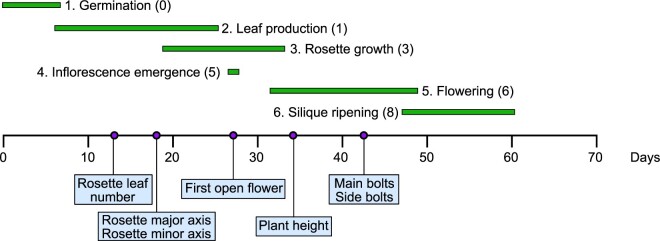
Distribution of observed traits in the growth of *A. thaliana* as adapted from Boyes et al. (2001). The purple dots along the *x*-axis represent the timing of phenotypic measurements. The number in parentheses indicates the growth stage, as defined by Boyes et al. (2001).

A set of seven morphological traits (the length of the rosette major axis, length of the rosette minor axis, leaf number, number of stem branches on main bolts, number of side bolts, time until the first open flower, and height of the main bolt at landmark growth stages) were collected (Figure 8). About 400 individuals of each genotype (WT; single T-DNA insertion lines and *exov exovl* double mutant lines) were grown in the greenhouse at the University of Chicago for observation of phenotypes; and 100 individuals of each of CRISPR/Cas9 lines and Col-0 were grown in soil-flats in the greenhouses at KIB. For the calculation of rosette axis and the number of rosette leaves, soil-grown plants at stage 1.04 (15 days) were measured with a vernier caliper, and leaves were counted. The time at which the first flower opened was scored between stage 3.00 (23 days) and stage 6.90 (50 days). In addition, the height of soil-grown plants at stage 6.10 (36 days) was measured with a vernier caliper and ruler, and the number of bolting shoots was counted (Supplemental Table S8). The analysis of Arabidopsis growth and development presented here provides a framework for identifying and interpreting phenotypic differences in plants resulting from genetic variation caused by mutations (Boyes et al., 2001).

### Estimating the phenotypic effects distribution of mutants

To estimate the distribution of the phenotypic effects of mutations on the trait, the phenotypes associated with the new and parental genes we analyzed. For analytical tractability, we adopted the models of Turelli (1984), Sawyer et al. (2003), and Jones et al. (2007) (Turelli, 1984; Sawyer et al., 2003; Jones et al., 2007), assuming that the phenotypic effects of mutant and WT alleles on a trait follow a Gaussian distribution:

$$f\left(x;\mu ,\sigma \right)={\left(2\pi \sigma^{2}\right)}^{-1/2}\cdot \exp(-{\left(x-\mu \right)}^{2}/2\sigma^{2})$$

with mean μ and standard deviation σ.

The distribution of mutational effects on each trait was inferred from the changes in the trait value among the mutants and the WT. Phenotypic differences in each of our seven traits between WT and mutant lines were assessed for both the T-DNA insertions and CRISPR/Cas9 mutants. Although the formal distribution of the mutational effects for any given trait is unknown, the change in the distribution of mutational effects on a trait can be inferred by the deviation from the distribution of trait value in the WT, such as a shift in the frequency peak. The theoretical curve for each of the observed trait distributions was determined as the best fitted curve of a Gaussian distribution using R (v4.0.4).

### Principal component analysis

Principal component analysis was performed on the seven morphological traits, using phenotypes measured on the T-DNA insertion lines and double mutant lines, CRISPR/Cas9 lines, and WT plants. Because the T-DNA insertion lines and the CRISPR knockout lines were grown in two separate experiments, they were considered separately. PCA was performed using the functions *predict()* and *princomp()* in R v4.0.4.

A technical issue is that the data have many data points (e.g. *exov* has more than 1,000 individuals of three mutants), which would make it hard to visualize the phenotypic differences of various mutants. We developed a simple geometric method to calculate the phenotypic distance between the new gene *EXOV* and the parental gene *EXOVL*, which are defined by the pairs of average principal components of each genotype. The first two principal components, PC1 and PC2, which are highly representative of the variation of morphological traits we investigated (∼80%), were used to form a 2D space. If we use Gi to denote a gene i in a pair of average PC values, PC1(Gi) and PC2(Gi), that are given by PCA for a population, then the difference in PED between the two genes can be mathematically described by using a geometric distance between gene mutants i and j measured by the following formula:

$$\text{PED}\;{\left(\text{Gi},\;\text{Gj}\right)}^{2}={\left[\text{PC}1\left(\text{Gi}\right)\;–\;\text{PC}1\left(\text{Gj}\right)\right]}^{2}+{\left[\text{PC}2{\left(\text{Gi}\right)}_{}–\;\text{PC}2\left(\text{Gj}\right)\right]}^{2}$$

giving,

$$\mathrm{PED}\;\left(\mathrm{Gi},\;\mathrm{Gj}\right)=\;\sqrt{{\left[\mathrm{PC}1\left(\mathrm{Gi}\right)–\;\mathrm{PC}1\left(\mathrm{Gj}\right)\right]}^{2}+{\left[\mathrm{PC}2\left(\mathrm{Gi}\right)–\;\mathrm{PC}2\left(\mathrm{Gj}\right)\right]}^{2}}.$$

Thus, the PED describes a distance of phenotypic evolution that occurs in the two genes in terms of eigenvectors of the measured morphological traits. This geometrical description is helpful when we compare the contribution of new gene and parental gene in a large data set of measured morphological traits.

### Sequence comparison of *EXOV* and *EXOVL*

Protein sequences for EXOV and EXOVL were downloaded from TAIR (http://www.arabidopsis.org/) and aligned by Geneious (Drummond et al., 2011). Orthologous coding sequences for *EXOVL* were downloaded from Phytozome v9.1 (http://www.phytozome.net/). Alignments of coding sequences mentioned below were performed by MEGA 5.2.2, considering the coding structures. For synteny analysis, genetic location information on *EXOV* and *EXOVL* were obtained from the TAIR website (http://www.arabidopsis.org/). The syntenic relationship among *EXOV*, *EXOVL*, and the orthologous genes Aly496175 (*A.* *lyrata*), Cru10026530 (*C.* *rubella*), Tha10013696m (*T.* *halophila*), Bra020254 (*B.* *rapa*), and Osa05g03200 (*Oryza sativa*) was displayed by Phytozome (http://www.phytozome.net/). For phylogenetic analysis, gene sequences of *EXOV* and *EXOVL* were aligned with *C.* *rubella*, *Eutrema salsugineum*, *Brassica*, and *Oryza* using Geneious and manually adjusted. A phylogenetic tree was created according to the maximum likelihood method using the MEGA 5.2.2 program (Tamura et al., 2011).

### Population genetics of *EXOV* and *EXOVL*

Genotypes of worldwide accessions were obtained from the Arabidopsis 1001 Genomes Project (Supplemental Data Set S1). This data set was used for population genetic analysis, including the 851 accessions that remained after and discarding sequences of poor quality or with sequencing errors (Anastasio et al., 2011). Basic population genetic analyses were implemented in the DnaSP5 program. Sequence diversity was calculated using nucleotide diversity (π) and the population mutation parameter of Watterson’s estimator. Synonymous substitution rates (Ks) and nonsynonymous substitution rates (Ka) were calculated using DnaSP5.10.1 (Rozas et al., 2003).

### Substitution analysis and testing selection

Following strict parsimony, all substitutions were identified that contribute to the divergence of *EXOV* and *EXOVL* and assigned to one of the two gene lineages following the duplication event. These analyses were conducted from a multiple gene sequence alignment (Supplemental Files S3 and S4), based on the states of the orthologs in outgroup species, defined by a phylogeny {[(*A. thaliana*, (*A. lyrata, A halleri*)), (*C. rubella, C. sativa*)], (*B. rapa and E. salsugineum*)} (genus names: *C., Capsella* or *Cannabis*; *B., Brassica; E., Eutrema*). All sites revealing substitutions on *EXOVL* before the duplication event were also counted. These sites were compared to the polymorphism tables from the 851 *A. thaliana* accessions, which produced 709 *EXOV* alleles and 455 *EXOVL* alleles. While most substitutions are present in 100% of the accessions, a few are present in ∼99% of alleles, with no ancestral alleles detected in the population. Tests of deviation from neutrality were conducted by comparing the observed substitutions with the polymorphisms at synonymous and nonsynonymous sites to test the distinctive prediction of neutral theory that the rates of mutation and evolution are equal, following a pipeline we designed for the algorithm (Supplemental Figure S9). In particular, the McDonald–Kreitman test was performed to detect positive selection acting on *EXOV* since its origination from the parental gene *EXOVL*.

## Supplemental data

The following materials are available in the online version of this article.

**Supplemental File S1.** SnapGeneViewer file for *EXOV*.

**Supplemental File S2.** Mapping the chromosomal insertion positions of the corresponding T-DNA lines of *EXOV* and *EXOVL*.

**Supplemental File S3**. Multiple sequence alignment used for substitution analysis and testing selection.

**Supplemental File S4.** Newick file format of the tree based on Supplemental File S2.

**Supplemental Data Set S1.** *Arabidopsis* *thaliana* accessions for population structure analysis.

**Supplemental Data Set S2.** Tajima’s *D* test, the Fay–Wu test, the Fu–Li tests across the Arabidopsis genome.

**Supplemental Data Set S3.** Differentially expressed genes between *exov-crp* and *exovl-crp*.

**Supplemental Data Set S4.** Differentially expressed genes between *exov* and *exovl*.

**Supplemental Table S1.** Substitution and polymorphism data for the McDonald–Kreitman test.

**Supplemental Table S2.** GO enrichment analysis of the set of genes that significantly differentially expressed between *exov* and *exovl.*

**Supplemental Table S3.** Pairwise comparisons for phenotypic traits of T-DNA mutants and CRISPR-Cas9 mutants (Wilcoxon rank sum test).

**Supplemental Table S4.** GO enrichment of analysis of significantly differentially expressed genes between the WT and *exovl* or WT and *exov*.

**Supplemental Table S5.** Primers for allele-specific PCR, RT-PCR, and RT-qPCR.

**Supplemental Table S6.** Identification of T-DNA insertion and CRISPR target site in the mutant lines by using whole-genome sequencing.

**Supplemental Table S7.** Mapping the off-target editing sites for *EXOV* and *EXOVL* in CRISPR knockout lines.

**Supplemental Table S8.** Phenotypic measurements for analysis.

**Supplemental Table S9.** Summary of sequencing data for RNA-seq.

**Supplemental Figure S1.** Target sites of the sgRNAs for At3G57110 (EXOV) and At5g60370 (EXOVL).

**Supplemental Figure S2 and S3.** Analyses of population structure for the world-wide accessions used in this study (the 1001 Genomes Project).

**Supplemental Figure S4.** Empirical distributions of several population genetic test parameters across the genome in *A. thaliana* and the probabilities of *EXOV* and *EXOVL* in these distributions.

**Supplemental Figure S5.** Protein sequence divergences of EXOVL and EXOV.

**Supplemental Figure S6.** eFP expression of *EXOV* (A) and *EXOVL* (B) in the WT Arabidopsis by Klepikova Arabidopsis Atlas (http://bar.utoronto.ca/eplant/).

**Supplemental Figure S7.** GO analyses.

**Supplemental Figure S8.** Distribution of the phenotypic effects on seven traits of T-DNA mutants lines (single *exov, exovl* and double *exov exovl*) and CRISPR/Cas9 mutant lines (*exov-crp exovl-crp*) of the new gene and parental gene and WT line (Col-0).

**Supplemental Figure S9.** Summary of neutrality test pipeline.

### Accession numbers

Accession numbers based on The Arabidopsis Information Resource (TAIR) (https://www.arabidopsis.org) for all genes examined in this study are *EXOV* (At3g57110), *EXOVL* (At5g60370). Sequence data for whole-genome resequencing are deposited in the NCBI database under BioProject number PRJNA766299. Sequence data for RNA-seq are deposited in the NCBI database under BioProject accession PRJNA766434.

## Supplementary Material

koab291_Supplementary_Data

## Notes

M.L., J.B., and Y.H. designed this research. Y.H. wrote the initial draft of the manuscript. Y.H. and J.C. performed all of the experiments involving the phenotypic observation and analysis, mutant generation, identification, expression, transcriptome and genome sequencing, and evolutionary analysis. C.D. and S.D. participated in the population genetic analysis. C.F. provided plant materials. C.F., Y.O., D.L., S.X., and E.M. revised the manuscript. M.L. and J.B. supervised the study, conceived and supervised the writing.

The author(s) responsible for distribution of materials integral to the findings presented in this article in accordance with the policy described in the Instructions for Authors (https://academic.oup.com/plcell) is: Manyuan Long (mlong@uchicago.edu).
