# Supplementary material for: Species-specific partial gene duplication in Arabidopsis thaliana evolved novel phenotypic effects on morphological traits under strong positive selection
Source: Plant Cell. 2021 Dec 7;34(2):802–17. doi: 10.1093/plcell/koab291 (PMC8824575; doi:10.1093/plcell/koab291)
Supplement: koab291_Supplementary_Data [file koab291_supplementary_data.zip › TPC2021RA00624R1 Supplemental Figures and Tables.pdf]

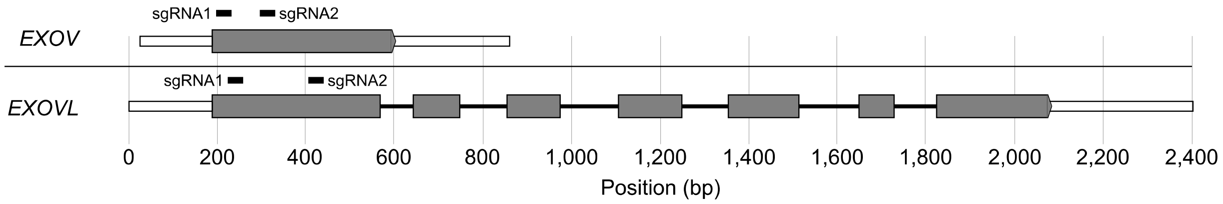

Supplemental Figure S1. Target sites of the sgRNAs for At3G57110 (*EXOV*) and At5g60370 (*EXOVL*). (Supports Figure 1).

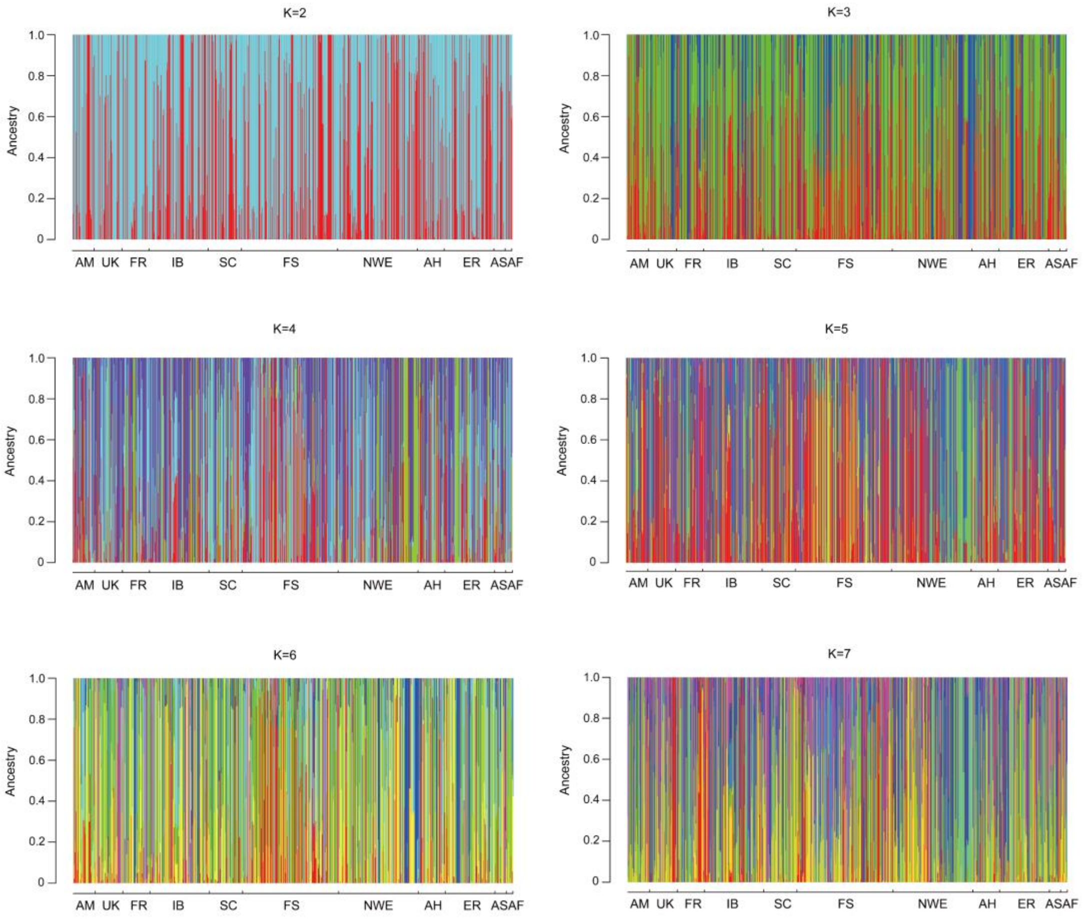

Supplemental Figure S2. Analyses of population structure for the worldwide accessions used in this study (the 1001 Genomes).
Population structure under different assumptions about the number of clusters (K=2, 3, 4, 5, 6, 7).
Supports Figure 2.

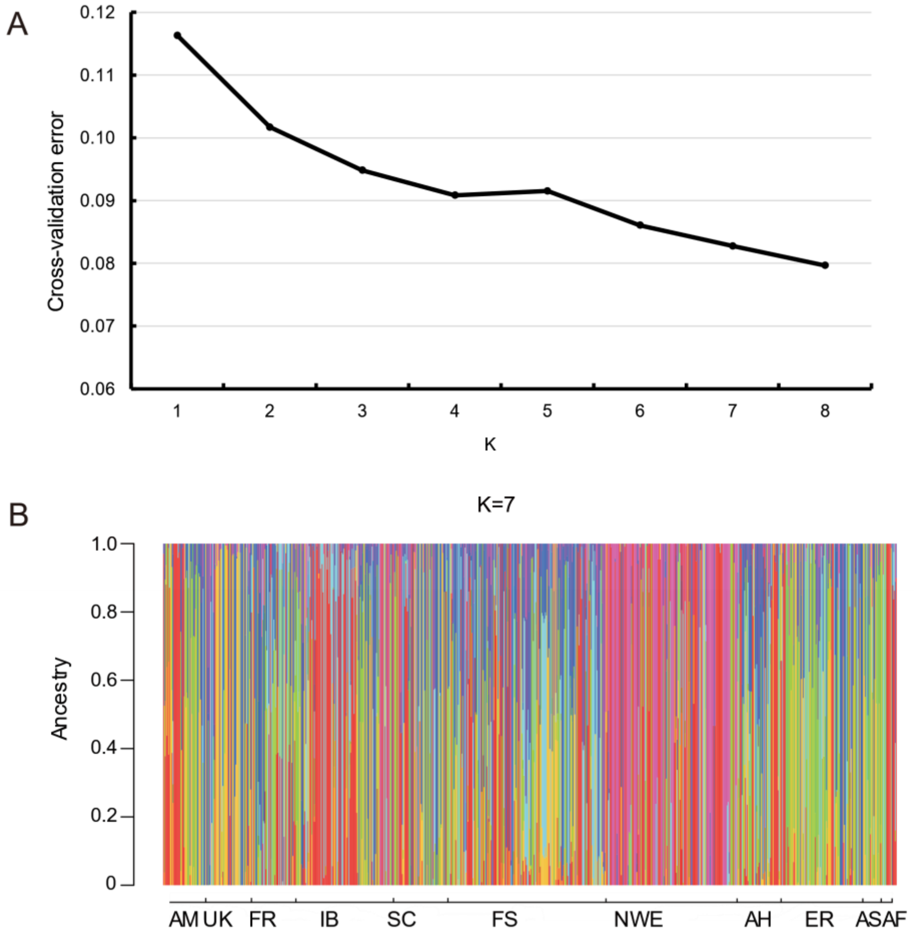

Supplemental Figure S3. Analyses of population structure for the worldwide accessions used in this study (the 1001 Genomes).
A. Cross-validation errors at various K values. **B.** Population structure analysis of worldwide 851 *A. thaliana* accessions (K = 8).
Supports Figure 2.

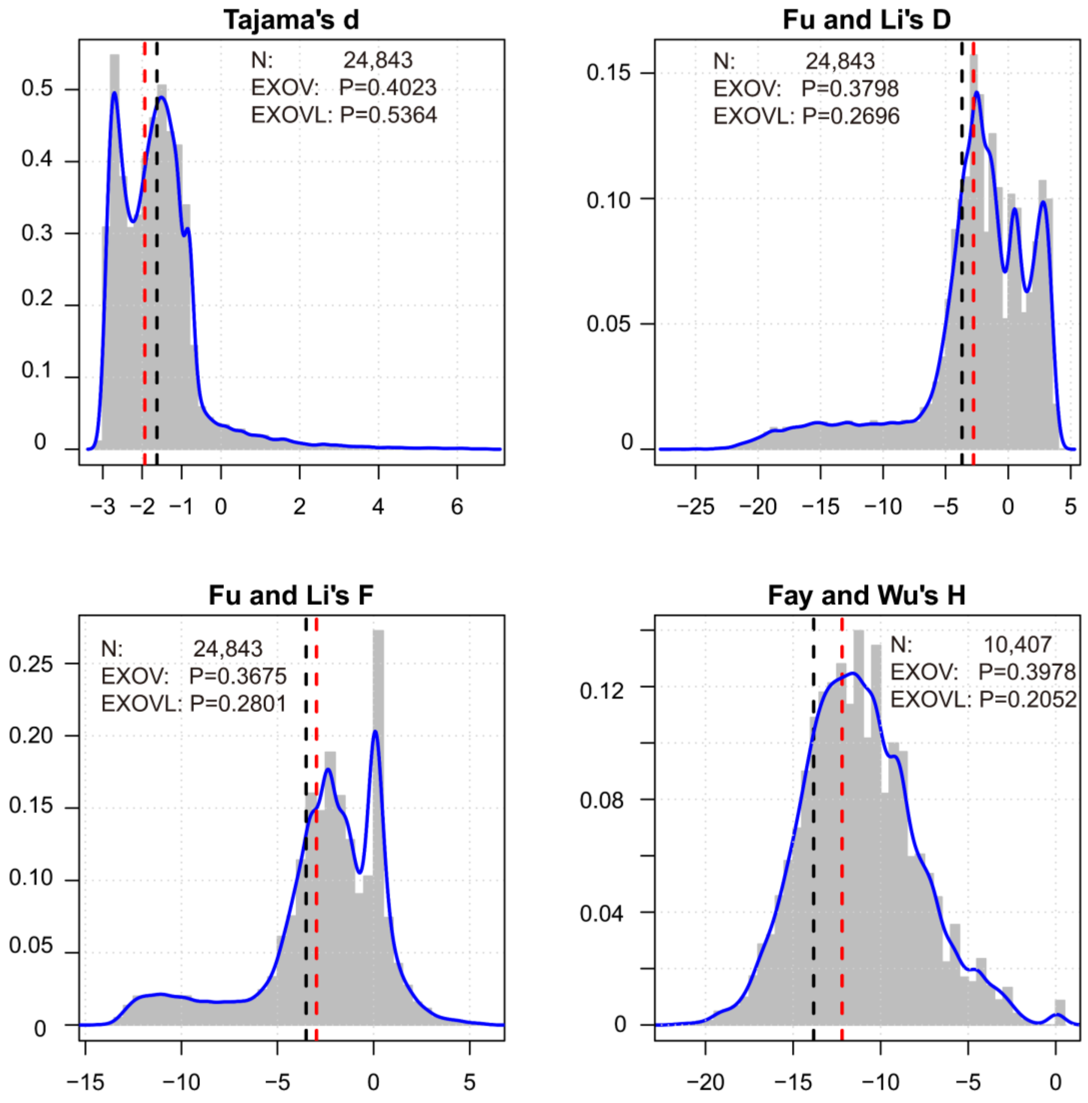

Supplemental Figure S4. Empirical distributions of several population genetic test parameters across genome in *A. thaliana* and the probabilities of *EXOV* and *EXOVL* in these distributions.
N: the number of genes used in the analysis. *P* is the probability equal to or lower than the observed parameters in *EXOV* (red vertical dashed line) or *EXOVL* (black vertical dashed line).
Supports Figure 2.

A

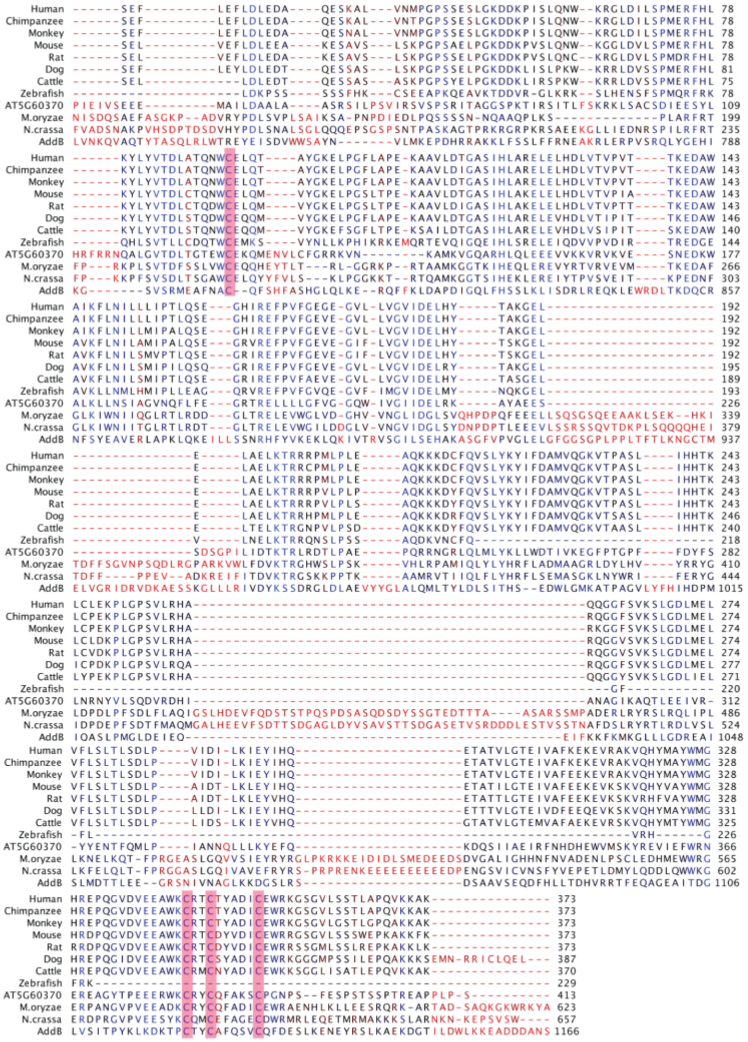

B

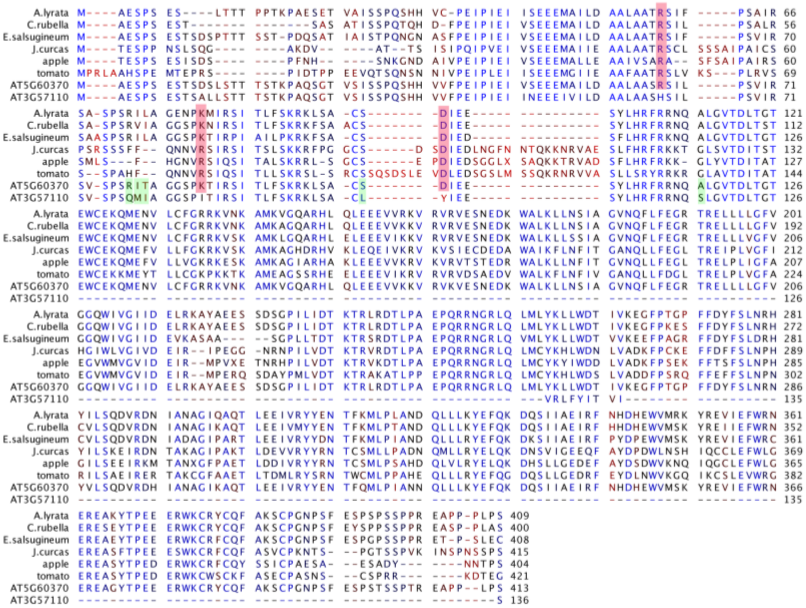

C

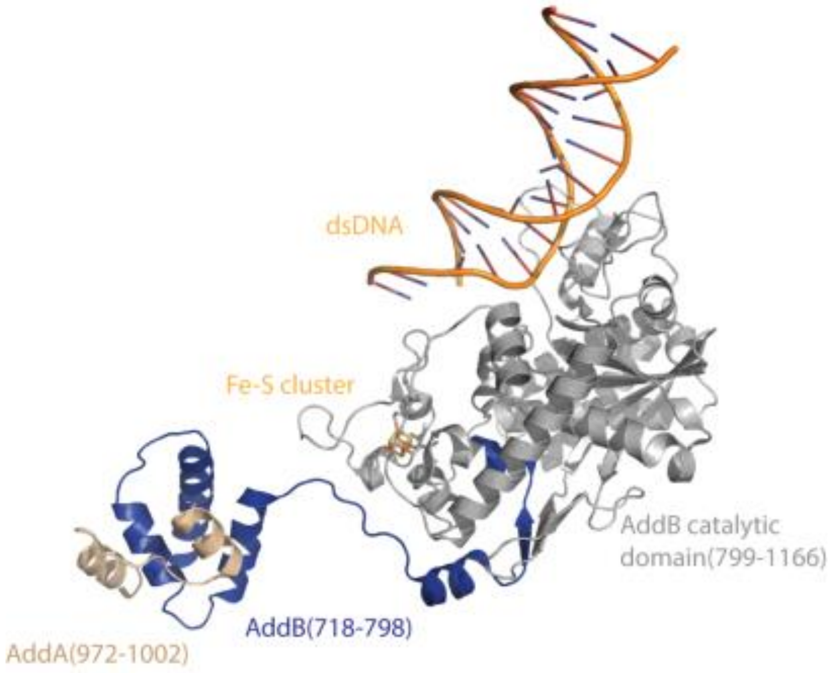

Supplemental Figure S5. Protein sequence divergences of EXOVL and EXOV.
A. Alignment of EXOVL with its homologs from different species and AddB (from *B.subtilis*). These homologs are from human (NP_073611.1), chimpanzee (XP_003308065.1), monkey (XP_001084006.1), mouse (NP_001153515.1), rat (NP_001101443.1), Dog (XP_532542.1), cattle (NP_001075077.1), zebrafish (NP_001032490.1), *M. oryzae* (XP_003718794.1), and *N. crassa* (XP_955908.1). The conserved cysteine residues that coordinate the Fe-S cluster are highlighted in red. **B**. Alignment of EXOV and EXOV-L with its plant orthologs. The conserved polar residues at positions 63, 85, and 103 of EXOV-L and its orthologs are highlighted in red. At position 63 of EXOV-L, the conserved residue is the basic polar residue arginine (R). In EXOV, this residue evolved to histidine (H). At position 85, the residue is either basic polar residue lysine (K) or arginine (R) in all instances except for that of EXOV, where it is substituted with the hydrophobic residue isoleucine (I). The conserved residue at position 103 is the acidic charged residue aspartate (D), which is changed to tyrosine (Y) in EXOV. Other residues such as R77, I78, T79, S102, and A119 were substituted with Q77, M78, I79, L102, and S119, highlighted in green. The NCBI accession numbers for the orthologs are XP_002864682 (*A. lyrata*), XP_006280574 (*C. rubella*), XP_006400854 (*E. salsugineum*), KDP44101 (*J. curcas*), XP_008358302 (apple), and XP_004251259 (tomato). **C.** Proposed structural model of EXOV-L showing its conservation.
Supports Figure 3.

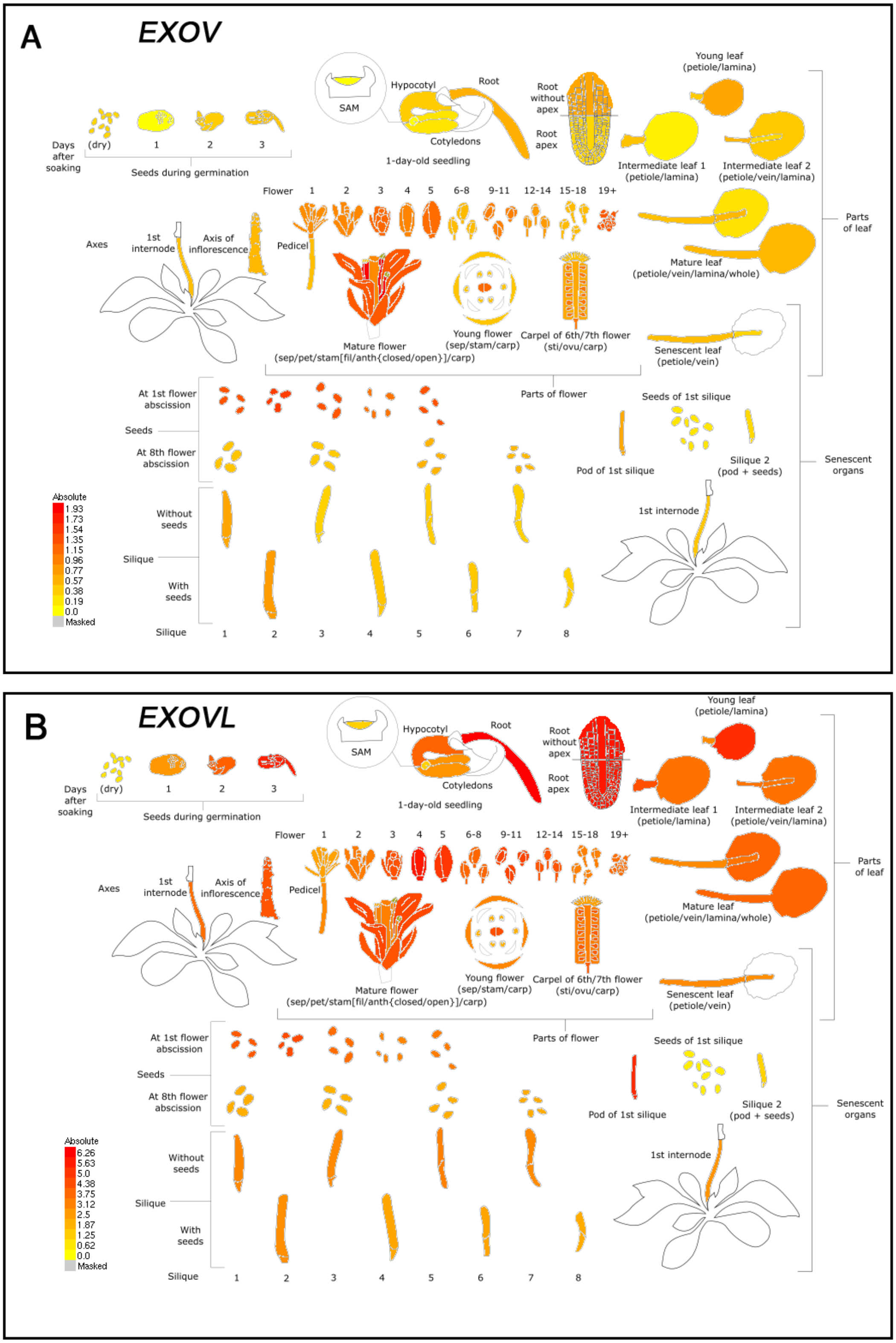

Supplemental Figure S6. eFP expression of *EXOV* (**A**) and *EXOVL* (**B**) in wild-type Arabidopsis based on the Klepikova Arabidopsis Atlas (http://bar.utoronto.ca/eplant/).
Supports Figure 3.

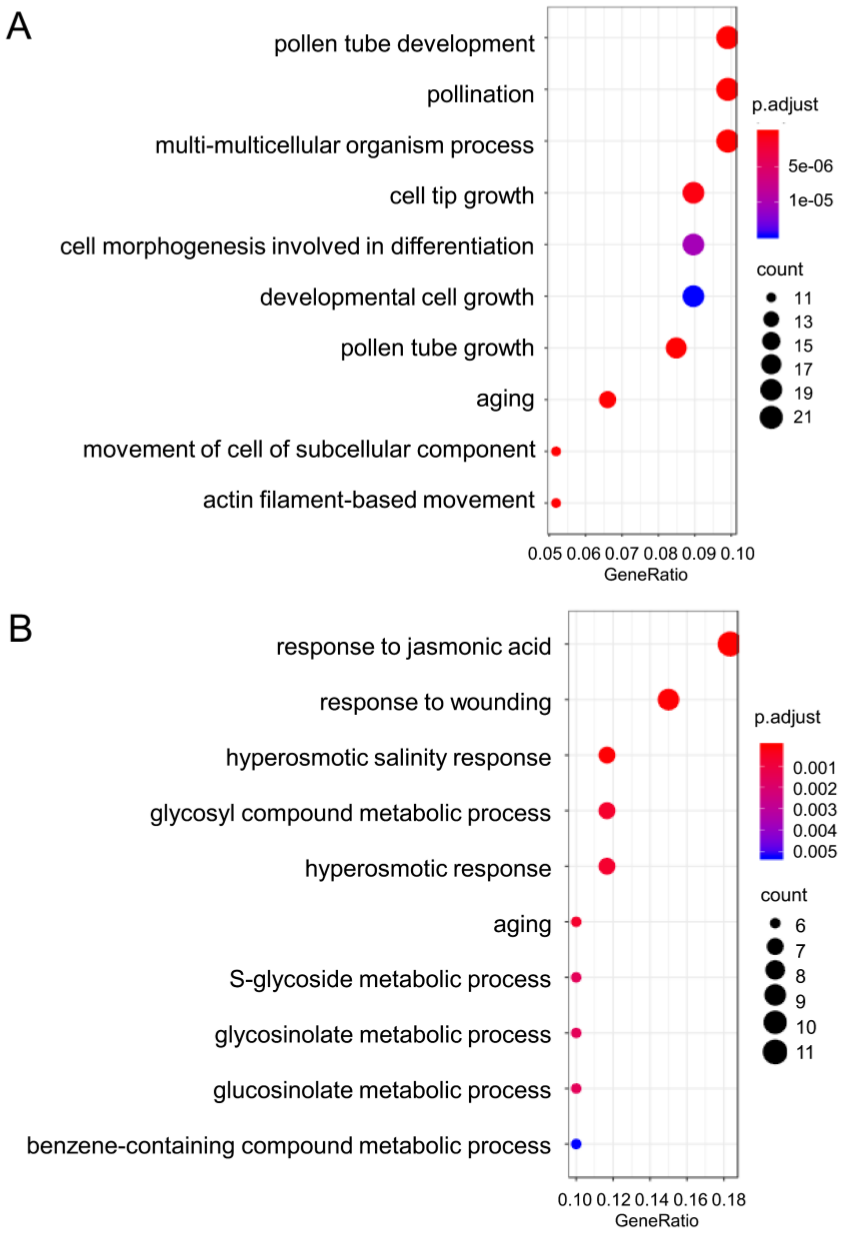

Supplemental Figure S7. GO analyses.
A. GO enrichment of analysis of differentially expressed genes between wild type and *exov-crp*. **B.** GO enrichment of analysis of differentially expressed genes between wild type and *exovl-crp.*
Supports Figure 3.

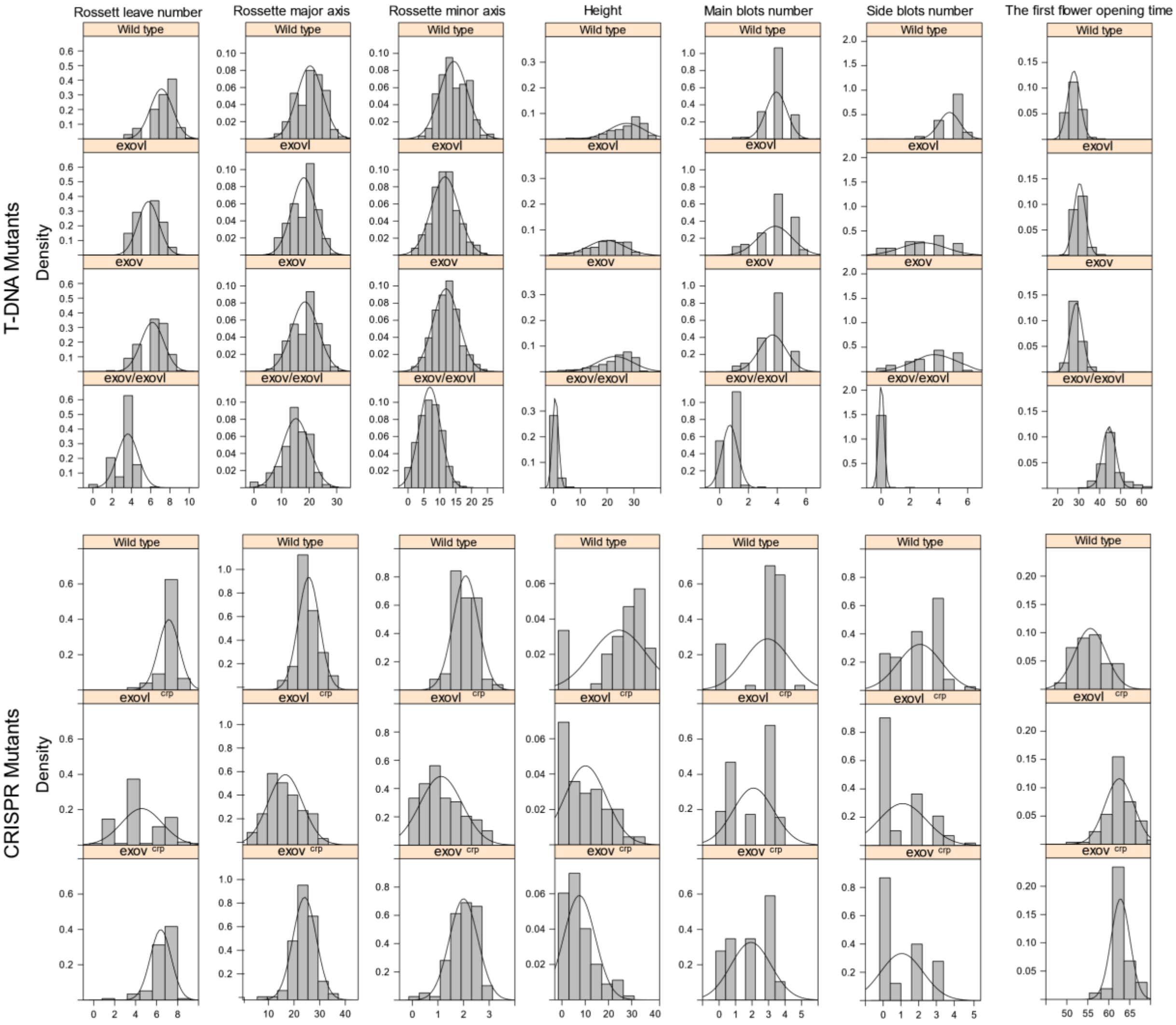

Supplemental Figure S8. Distribution of the phenotypic effects on seven traits of T-DNA mutants lines (single *exov-1, exovl-1* and the double mutant *exov-1 exovl-1*) and CRISPR/Cas9 mutant lines (*exov-crp*, *exovl-crp*) and wild-type (Col-0). The curves are theoretical distributions modeled as a Gaussian distribution.
Supports Figure 5.

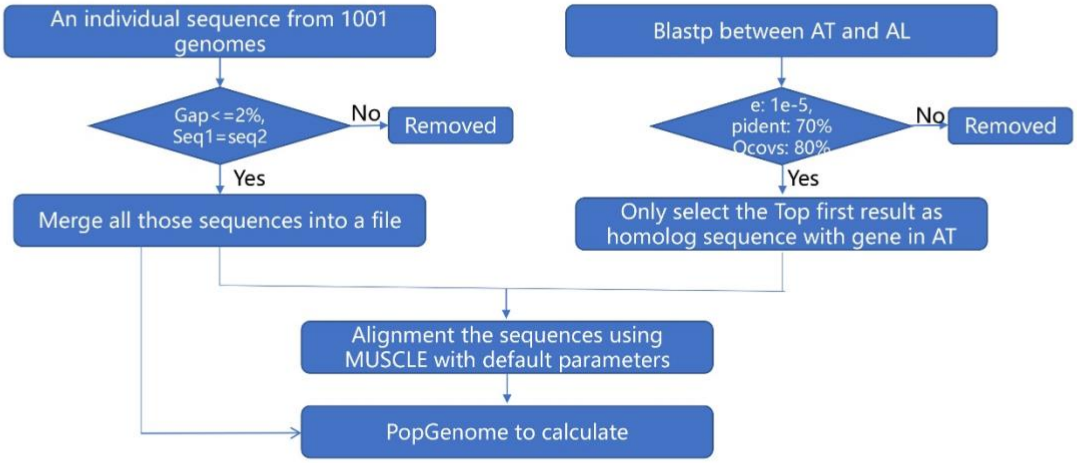

Supplemental Figure S9. Summary of neutrality test pipeline.

Supplemental Tables.

Supplemental Table S1. Substitution and polymorphism data for the McDonald-Kreitman test.

A. Substitution sites.

The substitutions are inferred from the parsimonious method taking *A. lyrata* as outgroup. The example below shows the divergence between *EXOV-L* and *EXOV* at site 120 as C and T, respectively. Thus, substitution at this site is parsimoniously inferred as C→T in the *EXOV* lineage (red) because the *EXOV-L* in *A. thaliana* and its ortholog in the outgroup *A. lyrata* are C at the site, which indicate that the two ancestral states are C.

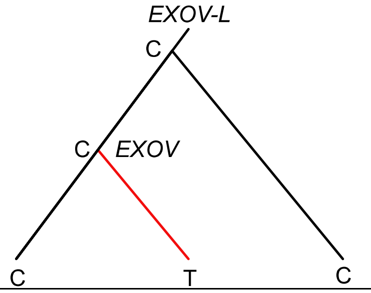

Synonymous substitutions.

EXOV	33G (x0.5), 120T, 207C, 210T, 225G, 252A, 276T, 339A, 348G, 375T,402C(x0.5), 408A, 198A	Sum: 12
EXOVL	249T, 255A, 264C, 375A	Sum: 4

Non-synonymous substitutions.

EXOV	32C(x1.5), 35T, 139A, 143A, 158T, 188A, 230A,254T, 305T, 307T, 349T, 380T, 385T, 395T, 398C, 400G, 401T (x1.5), 404T, 156A, 234G, 255A	Sum: 22
EXOVL	184T, 206T, 236C	Sum: 3

B. Polymorphisms

Synonymous polymorphisms (gene/accession numbers, proportion for nucleotide)

Sites	117	183	225	282	312	339	348	351	360	363	375	411
EXOV-709	0.990C 0.010T	0.997C 0.003T		0.993C 0.007T	0.032A 0.968C			0.999T 0.001C	0.993C 0.007T	0.994A 0.006G	0.024C 0.976T	0.001A 0.999G
EXOVL-455			0.154A 0.846G			0.402A 0.598G	0.613A 0.387G				0.730A 0.270C	

Synonymous polymorphisms (gene/accession numbers, proportion for nucleotide)

Sites	64	88	139	143	163	202	205	296	307	316
EXOV-709				0.863A 0.137G					0.103C 0.897T	0.102A 0.898G
EXOVL-455	0.035A 0.965G	0.026G 0.974T	0.515A 0.485G		0.967C 0.033A	0.532A 0.468T	0.035A 0.965G	0.974C 0.026G		

Supplemental Table S2. GO enrichment analysis of differentially expressed genes between *exov* and *EXOV-L*.

GO Term	Count	%	P-Value	Benjamini
Biosynthesis of secondary metabolites	40	6.6	$5.70x10^{-05}$	$1.90x10^{-03}$
2-Oxocarboxylic acid metabolism	7	1.1	$3.60x10^{-03}$	$4.80x10^{-02}$
Plant hormone signal transduction	12	2	$1.70x10^{-02}$	$1.60x10^{-01}$

Supplemental Table S3. Pairwise comparisons for phenotypic traits of T-DNA mutants and CRISPR-Cas9 mutants (Wilcoxon rank sum test).

T-DNA insertion lines

		exov	*exovl*	*exov exovl*
Leaf number	WT	$< 2\times10^{-16}$	$< 2\times10^{-16}$	$< 2\times10^{-16}$
	exov		1.90×10^{-10}	$< 2\times10^{-16}$
	exovl			$< 2\times10^{-16}$
Flowering time	WT	1.0×10^{-11}	$< 2\times10^{-16}$	$< 2\times10^{-16}$
	exov		$< 2\times10^{-16}$	$< 2\times10^{-16}$
	exovl			$< 2\times10^{-16}$
Height	WT	$< 2\times10^{-16}$	$< 2\times10^{-16}$	$< 2\times10^{-16}$
	exov		$< 2\times10^{-16}$	$< 2\times10^{-16}$
	exovl			$< 2\times10^{-16}$
Number of branches on main bolt	WT	2.50×10^{-05}	0.6145	$< 2\times10^{-16}$
	exov		0.0001	$< 2\times10^{-16}$
	exovl			$< 2\times10^{-16}$
Number of branches on side bolts	WT	$< 2\times10^{-16}$	$< 2\times10^{-16}$	$< 2\times10^{-16}$
	exov		4.70×10^{-08}	$< 2\times10^{-16}$
	exovl			$< 2\times10^{-16}$
Rosette major axis	WT	5.20×10^{-10}	3.60×10^{-11}	$< 2\times10^{-16}$
	exov		0.096	$< 2\times10^{-16}$
	exovl			$< 2\times10^{-16}$
Rosette minor axis	WT	$< 2\times10^{-16}$	$< 2\times10^{-16}$	$< 2\times10^{-16}$
	exov		0.088	$< 2\times10^{-16}$
	exovl			$< 2\times10^{-16}$

CRISPR-Cas9 mutants

		exov-crp	*exovl-crp*
Leaves number	WT	7.60×10^{-07}	4.70×10^{-16}
	exov-crp		5.60×10^{-12}
Flowering time	WT	$< 2\times10^{-16}$	$< 2\times10^{-16}$
	exov-crp		0.61
Height	WT	2.60×10^{-16}	7.10×10^{-06}
	exov-crp		0.38
Number of branches on main bolt	WT	2.50×10^{-05}	0.6145
	exov-crp		0.0001
Number of branches on side bolts	WT	1.10×10^{-06}	3.30×10^{-06}
	exov-crp		0.97
Rosette major axis	WT	0.036	9.80×10^{-15}
	exov-crp		7.60×10^{-14}
Rosette minor axis	WT	0.55	1.00×10^{-11}
	exov-crp		1.10×10^{-12}

Supplemental Table S4. GO enrichment analysis of significantly differentially expressed genes between wild type and *exovl* or wild type and *exov*.

Wild type vs *EXOVL*

GO Term	Count	%	*P*-Value	Benjamini
Biosynthesis of secondary metabolites	39	8.5	$3.80x10^{-07}$	$2.50x10^{-05}$
Phenylpropanoid biosynthesis	14	3.1	$1.30x10^{-06}$	$4.20x10^{-05}$
Metabolic pathways	45	9.8	$2.40x10^{-03}$	$5.10x10^{-02}$
Phenylalanine metabolism	5	1.1	$4.80x10^{-03}$	$7.60x10^{-02}$
Glucosinolate biosynthesis	3	0.7	$1.60x10^{-02}$	$1.90x10^{-01}$
2-Oxocarboxylic acid metabolism	5	1.1	$3.30x10^{-02}$	$3.10x10^{-01}$
Cutin, suberine and wax biosynthesis	3	0.7	$7.20x10^{-02}$	$5.10x10^{-01}$
Cyanoamino acid metabolism	4	0.9	$7.60x10^{-02}$	$4.80x10^{-01}$
Stilbenoid, diarylheptanoid and gingerol biosynthesis	4	0.9	$7.90x10^{-02}$	$4.50x10^{-01}$
Indole alkaloid biosynthesis	2	0.4	$9.50x10^{-02}$	$4.80x10^{-01}$

Wild type vs *exov*

GO Term	Count	%	*P*-Value	Benjamini
Pentose and glucuronate interconversions	8	1.3	$1.20x10^{-03}$	$2.60x10^{-02}$
Alanine, aspartate and glutamate metabolism	4	0.7	$7.30x10^{-02}$	$4.00x10^{-01}$
Valine, leucine and isoleucine biosynthesis	3	0.5	$7.80x10^{-02}$	$3.90x10^{-01}$
Phenylalanine metabolism	6	1	$1.50x10^{-03}$	$2.40x10^{-02}$
Nitrogen metabolism	4	0.7	$5.30x10^{-02}$	$3.30x10^{-01}$
Phenylpropanoid biosynthesis	16	2.6	$4.30x10^{-07}$	$2.90x10^{-05}$
Glucosinolate biosynthesis	3	0.5	$2.40x10^{-02}$	$1.80x10^{-01}$
Metabolic pathways	52	8.5	$5.60x10^{-03}$	$6.10x10^{-02}$

Supplemental Table S5. Primers for allele-specific PCR, RT-PCR and RT-qPCR.

Primer name	Line or gene	Sequence (5' to 3')	amplicon size
Genotyping for T-DNA insertion lines			
AT3G57110-LP<br>AT3G57110-RP	SALK_101821	TAGCAAATTGGCAATACCGAC<br>AGCTGTTGAATTCCATTGCTG	810 bp
AT5G60370-1LP<br>AT5G60370-1RP	SALK_103969	GAAAAATTAGTCAGCAGTCGGG<br>CAATCATGGTGAGATTCCAAAG	1,201 bp
AT5G60370-2LP<br>AT5G60370-2RP	SALK_036494	CTCTCACAATTAGCCGCTGTC<br>TTGGAGAAATCATGGAGATCG	919 bp
AT5G60370-3LP<br>AT5G60370-3RP	SALK_064431	TGGAAGACGAAGTGGTAGGTG<br>CGTCGTCGCTACTATTCGATC	1,082 bp
LBb1.3		ATTTTGCCGATTTCGGAAC	
Genotyping CRISPR alleles			
AT3G57110-F<br>AT3G57110-R	*exov-crp*	GCATAGACATGAAAAAAGAAGAA<br>TTCCTATGATATGACTGTGATATA	
AT5G60370-F<br>AT5G60370-R	*exovl-crp*	GCTTTATTGACTTTTCTCCTGCCA<br>CACATGTTGGTTCCGAATAAAACA	
RT-PCR			
AT3G57110-1S<br>AT3G57110-1R	*EXOV*	ATTTCAAGCCCTCAAAGTCA<br>CAAACGTACAGTACCGGTGA	303 bp
AT3G57110-2S<br>AT3G57110-2R	*EXOV*	GTCACCCTCCGAGTCAACCT<br>AAACAAGCTGATAATTTCCT	297 bp
AT5G60370-1S<br>AT5G60370-1R	*EXOVL*	TTAGCACCACCACATCCACT<br>AATGACGAGCCTGACCAACT	429 bp
TUB2-F<br>TUB2-S	*TUBULIN 2*	GTTCTCGATGTTGTTCGTAAG<br>TGTAAGGCTCAACCACAGTAT	205 bp
Oligo(dT)20		TTTTTTTTTTTTTTTTTTTT	
RT-qPCR			
AT3G57110-qF<br>AT3G57110-qR	*EXOV*	CATCCACTAAGCCCGCTCAA<br>TGCAAGAGCTGCATCGAGAA	131 bp
AT5G60370-qF<br>AT5G60370-qR	*EXOVL*	CTTCCCCGAGATCCCAATCG<br>ATCACGGAAGGGAGGATGGA	96 bp
Act8-qRT-F<br>Act8-qRT-R	*ACTIN 8*	TCAGCACTTTCCAGCAGATG<br>CTGTGGACAATGCCTGGAC	70 bp

Supplemental Table S6. Identification of T-DNA insertion and CRISPR target site in the mutant lines by using whole-genome sequencing.

Gene	Mutant	Type	Total sites	Sequenced sites	Coverage	Depth	Insertion	On-target	Off-target
EXOV	SALK-103969-4	T-DNA	119,667,750	119,455,951	99.82%	67	C297720 3_21134853_21136786	NA	NA
EXOVL	60370-4	CRISPR	119,667,750	119,452,948	99.82%	56	NA	yes	no
EXOV	57110-4	CRISPR	119,667,750	119,450,723	99.82%	80	NA	yes	no
EXOVL	SALK-101821	T-DNA	119,667,750	119,450,151	99.82%	90	scaffold12830 5_24283931_24291840	NA	NA
EXOV	57110-1-15	CRISPR	119,667,750	119,089,802	99.52%	51	NA	yes	no
EXOV	SALK-103969-60	T-DNA	119,667,750	119,388,798	99.77%	73	C276322 3_21134853_21136020	NA	NA

Supplemental Table S7. Mapping the off-target editing sites for *EXOV* and *EXOV-LL* in CRISPR knockout lines.

sgRNA1 *EXOV* (5' TGTGGTGGTGCTAAGCAACG CGG 3')

Sequence	Off-score	# MM	Position	Gene	Region
TGTGATGGTGCGGAGCAACCGGGG	0.121	4	5 – (19,295,886)	At5g47580	exon
AGTGGTGGTGCAAAGTGACGAGG	0.043	4	4 – (4,435,296)	At4g07650	exon
TGTGGTGGTGCTAAGCGAG TCGG	0.015	3	5 – (24,282,892)	At5g60370	exon

sgRNA2 *EXOV* (5' TCTTCATTGATGATCTCGAT TGG 3')

Sequence	Off-score	# MM	Position	Gene	Region
ACTTCATCGACGATCTCGATCGG	0.667	3	4 + (2,804,545)	At4g05520	exon
TCTTCACTGACGATCTCGATTGG	0.458	2	5 – (24,282,990)	At5g60370	exon
CCTTGATTGATAATCTCCATTGG	0.229	4	2 + (13,716,797)	At2g32290	exon
GCTTCATGAATGATCTCCATCGG	0.215	4	3 – (357,834)	At3g02060	exon
TCTTCATTGATAATCTAATTTGG	0.162	4	3 + (6,226,936)		intergenic
TCTTGAATGATGAACTTGATGGG	0.152	4	1 – (26,432,964)	At1g70200	exon
TCATCCTTGGAGATCTCGATCGG	0.135	4	5 + (26,012,207)	At5g65110	exon
TCTCCATTGGTCATCTCCATTGG	0.091	4	1 + (21,640,530)		intergenic
TCTTCATTGATAAAATCAATAGG	0.089	4	1 + (5,604,058)	At1g16390	exon
TCTTCTTTGGTGATCTTCATCGG	0.088	4	3 – (7904408)	At3g22360	edon
TCTTGATGGATGCTCACGATTGG	0.084	4	5 – (6080618)	At5g18360	exon
TTATCACTAATGATCTCGATTAG	0.074	4	1 + (7800235)		intergenic
TCTTCTTCGATGATCTCTGTTAG	0.046	4	3 – (21528910)	At3g58130	exon
TCTTCTTCGATGATCTCTGTTAG	0.046	4	2 – (11698357)	At2g27340	exon
TCTTCATTTCTGATATTGATAGG	0.018	4	2 + (634172)	At2g02410	intron
TCTTCATTGATGTTTTCCATAGG	0.010	3	3 + (5016598)	At3g14910	intron
TTTACATTGATGATGTTGATTGG	0.010	4	5 + (1076795)	At5g03990	exon
TCTTCTTTAATTATGTCGATGGG	0.009	4	2 – (1822137)		intergenic
TCTTCTTTAATTATGTCGATGGG	0.009	4	4 – (5331905)	At4g08406	CDS
TCTTCATTGATGAGCTGCTTGGG	0	4	3 + (17335888)	At3g47060	exon

sgRNA1 *EXOVL* (5' GGTGGTGCTAAGCGAGTCGG AGG 3')

Sequence	Off-score	# MM	Position	Gene	Region
GAAGGTGCTAAGCGAGTCTTCGG	0.282	4	4 + (18048611)	At4g38600	exon
GGTGATGCTAACCGAGGTGGAGG	0.035	4	2 – (14039782)	At2g33100	exon
GGTGGTGCTAAGCAACGCGGAGG	0	3	3 – (21134955)	At3g57110	CDS
GGTGGTGCTAAACGACACGGAAG	0	3	2 + (1033750)	At2g03410	UTR

sgRNA2 *EXOVL* (5' CCGCCGGCGGTTATCCGTGA CGG 3')

Sequence	Off-score	# MM	Position	Gene	Region
CCGTCGGAGGTTATCAGTGGGGG	0.398	4	5 + (7136245)	At5g21010	exon
CCGCCGGCGATCATCTGTG	0.272	4	3 – (21135152)	At3g57110	CDS

MM, number of mismatches. CDS, coding sequence. UTR, untranslated region.
+, sense strand. –, antisense strand.

Supplemental Table S8. Phenotypic measurements for analysis.

Measurement	Unit	Growth stage	Description
number of rosette leaves	count	1.04	4 rosette leaves > 1 mm in length
rosette major axis	mm	1.08	8 rosette leaves > 1 mm in length
rosette minor axis	mm	1.08	8 rosette leaves > 1 mm in length
Height	mm	6.1	10% flower to be produced have opened
Bolt	count	6.1	10% flowers have opened<br>• Main bold number<br>• Side bolt number
First flower open	day	6	First flower open

Supplemental Table S9. Summary of sequencing data for RNA-seq.

	**Number of read (million)**		
Genotype	Replicate 1	Replicate 2	Replicate 3
Col-0	45.3	58.6	57.4
exov	62.3	51.4	58.5
EXOVL	62.9	68.8	65.3
Col-0	71.3	68.1	72.1
exov-crp	69.1	69.7	74.1
exovl-crp	67.2	71.4	71.9
