# Supplementary material for: Species-specific partial gene duplication in Arabidopsis thaliana evolved novel phenotypic effects on morphological traits under strong positive selection
Source: Plant Cell. 2021 Dec 7;34(2):802–17. doi: 10.1093/plcell/koab291 (PMC8824575; doi:10.1093/plcell/koab291)
Supplement: koab291_Supplementary_Data [file koab291_supplementary_data.zip › TPC2021RA00624R1 Supplemental File S2.pdf]

Supplemental File S2. Mapping the chromosomal insertion positions of the corresponding T-DNA lines in *EXOV* and *EXOVL.*

https://blast.ncbi.nlm.nih.gov/Blast.cgi?PAGE_TYPE=BlastSearch&SEARCH_INIT=Repr Genom eDBSearch&TAXID=3702

Sequence-103969-32 (At3g57110)

```
CGTCATGTAGATACCTAACTTAGATGCGTCTCACTGGTGAAAAGAAAAACCACCCCAGTACATTAAAAA
CGTCCGCAATGTGTTATTTTACTCTGGTTTGAATTTTTTGCATAGACATGAAAAAAGAAGAAGACCACA
CGTTGGCTCCGAATAAAACATGGCCGAGTCACCCTCCGAGTCAACCTCCGCGTTGCTTAGCACCACCAC
ATCCACTAAGCCCGCTCAATCCGGGACTGTTTCTATTTCAAGCCCTCAAAGTCACCATGTCGTCTTCCC
TGAGATCCCAATCGAGATCATCAATGAAGAAGAAATAGTTATTCTCGATGCAGCTCTTGCAGCCTCCCA
CTCCATCCTACCTTCCGTCATTCGTTCTGTTTCTCCGTCACAGATGATCGCCGGCGGAAGCCCAATAAC
TATTCGTTCAATTACTCTTTTCTCCAAGAGGAAATTATCAGCTTGTTTATATATCGAAGAGTCTTATCT
ACATCGATTTAGAAGAAATCAGTCTTTAGGTGTCACAGATCTCACCGGTACTGTACGTTTGTTTTATAT
CACAGTCATATCATAGGAAGAAGAGATCGAATAGTAGCGACGACGTGGACAGCGGCTAATTGTGAGAGA
TGATTTAGATTTAGTACAATTGTTAGGTTTAATATCTCTTTTTCTGGTGAGATTAATTTGTGGTCCACT
ACTCATCCATTTCATTAATTTTCCATTTCATTGCTCATGTTATTTCATAATCTACAAACATATCTTTGG
AATCTCACCATGATTGATAATTTCATTAATTCCAAAAAATTAGAACTAAAGTTTTATCATAGAATTTAT
AAAATTTGTTGTTTTGGGGTTTCCTTAATATTACCTAATTTTTTAAG
```

Arabidopsis thaliana chromosome 3 sequence

Sequence ID: NC_003074.8 Length: 23459830 Number of Matches: 1

Range 1: 21134854 to 21135628 GenBank Graphics ▼ Next Match ▲ Previous Match

Score	Expect	Identities	Gaps	Strand
1395 bits(755)	0.0	775/783(99%)	8/783(1%)	Plus/Plus

Features: exonuclease V

```
Query  88        TTACTCTGGTTTGAATTTTTTGCATAGACATGAAAAAAGAAGAAGACCACACGTTGGCTC  147
                 ||||||||||||||||||||||||||||||||||||||||||||||||||||||||||||
Sbjct  21134854  TTACTCTGGTTTGAATTTTTTGCATAGACATGAAAAAAGAAGAAGACCACACGTTGGCTC  21134913

Query  148       CGAATAAAACATGGCCGAGTCACCCTCCGAGTCAACCTCCGCGTTGCTTAGCACCACCAC  207
                 ||||||||||||||||||||||||||||||||||||||||||||||||||||||||||||
Sbjct  21134914  CGAATAAAACATGGCCGAGTCACCCTCCGAGTCAACCTCCGCGTTGCTTAGCACCACCAC  21134973

Query  208       ATCCACTAAGCCCGCTCAATCCGGGACTGTTTCTATTTCAAGCCCTCAAAGTCACCATGT  267
                 ||||||||||||||||||||||||||||||||||||||||||||||||||||||||||||
Sbjct  21134974  ATCCACTAAGCCCGCTCAATCCGGGACTGTTTCTATTTCAAGCCCTCAAAGTCACCATGT  21135033

Query  268       CGTCTTCCCTGAGATCCCAATCGAGATCATCAATGAAGAAGAAATAGTTATTCTCGATGC  327
                 ||||||||||||||||||||||||||||||||||||||||||||||||||||||||||||
Sbjct  21135034  CGTCTTCCCTGAGATCCCAATCGAGATCATCAATGAAGAAGAAATAGTTATTCTCGATGC  21135093

Query  328       AGCTCTTGCAGCCTCCCACTCCATCCTACCTTCCGTCATTCGTTCTGTTTCTCCGTCACA  387
                 ||||||||||||||||||||||||||||||||||||||||||||||||||||||||||||
Sbjct  21135094  AGCTCTTGCAGCCTCCCACTCCATCCTACCTTCCGTCATTCGTTCTGTTTCTCCGTCACA  21135153

Query  388       GATGATCGCCGGCGGAAGCCCAATAACTATTCGTTCAATTACTCTTTTCTCCAAGAGGAA  447
                 ||||||||||||||||||||||||||||||||||||||||||||||||||||||||||||
Sbjct  21135154  GATGATCGCCGGCGGAAGCCCAATAACTATTCGTTCAATTACTCTTTTCTCCAAGAGGAA  21135213

Query  448       ATTATCAGCTTGTTTATATATCGAAGAGTCTTATCTACATCGATTTAGAAGAAATCAGTC  507
                 ||||||||||||||||||||||||||||||||||||||||||||||||||||||||||||
Sbjct  21135214  ATTATCAGCTTGTTTATATATCGAAGAGTCTTATCTACATCGATTTAGAAGAAATCAGTC  21135273

Query  508       TTTAGGTGTCACAGATCTCACCGGTACTGTACGTTTGTTTTATATCACAGTCATATCATA  567
                 ||||||||||||||||||||||||||||||||||||||||||||||||||||||||||||
Sbjct  21135274  TTTAGGTGTCACAGATCTCACCGGTACTGTACGTTTGTTTTATATCACAGTCATATCATA  21135333

Query  568       GGAAGAAGAGATCGAATAGTAGCGACGACGTGGACAGCGGCTAATTGTGAGAGATGATTT  627
                 ||||||||||||||||||||||||||||||||||||||||||||||||||||||||||||
Sbjct  21135334  GGAAGAAGAGATCGAATAGTAGCGACGACGTGGACAGCGGCTAATTGTGAGAGATGATTT  21135393
```

Sequence-103696-60 (At3g57110)

AGATACGTCTCACTGGTGAAGAAAACCACCCCAGTACATTAAAAACGTCCGCAATGTGTTATTTTACTC
TGGTTTGAATTTTTTGCATAGACATGAAAAAAGAAGAAGACCACACGTTGGCTCCGAATAAAACATGGC
CGAGTCACCCTCCGAGTCAACCTCCGCGTTGCTTAGCACCACCACATCCACTAAGCCCGCTCAATCCGG
GACTGTTTCTATTTCAAGCCCTCAAAGTCACCATGTCGTCTTCCCTGAGATCCCAATCGAGATCATCAA
TGAAGAAGAAATAGTTATTCTCGATGCAGCTCTTGCAGCCTCCCACTCCATCCTACCTTCCGTCATTCG
TTCTGTTTCTCCGTCACAGATGATCGCCGGCGGAAGCCCAATAACTATTCGTTCAATTACTCTTTTCTC
CAAGAGGAAATTATCAGCTTGTTTATATATCGAAGAGTCTTATCTACATCGATTTAGAAGAAATCAGTC
TTTAGGTGTCACAGATCTCACCGGTACTGTACGTTTGTTTTATATCACAGTCATATCATAGGAAGAAGA
GATCGAATAGTAGCGACGACGTGGACAGCGGCTAATTGTGAGAGATGATTTAGATTTAGTACAATTGTT
AGGTTTAATATCTCTTTTTCTGGTGAGATTAATTTGTGGTCCACTACTCATCCATTTCATTAATTTTCC
ATTTCATTGCTCATGTTATTTCATAATCTACAAACATATCTTTGGAATCTCACCATGATTGATAATTTC
ATTAATTCCAAAAATTAGAACTAAAGTTTTATCATAGAATTTATAAAATTTGTTGTTTGGGTTTCTTAT
ATACTATTTTAGGGTTTAGTTTTCCTAATTAAAAACAACTAAAAATCTTATAGAAATATATAAATTGGA
AAAGTTGTTGACTTTGAATCATTTGTGGAGAAATATCGCCTCTTCTATAGTCTGAAAATTTTAGCCGGC
GATGTATACTAACTCATCAGTCATCACAAATAGTAACGACTTTATACAAATAGTATACTATTTGATAAT
GGAATGCTCTCTTCCTTGCGCATCTTCATTTATGT

Arabidopsis thaliana chromosome 3 sequence

Sequence ID: NC_003074.8 **Length:** 23459830 **Number of Matches:** 1

Range 1: 21134854 to 21135860 GenBank Graphics ▼ Next Match ▲ Previou

Score	Expect	Identities	Gaps	Strand
1855 bits(1004)	0.0	1006/1007(99%)	0/1007(0%)	Plus/Plus

```
Query  64        TTACTCTGGTTTGAATTTTTTGCATAGACATGAAAAAAGAAGAAGACCACACGTTGGCTC  123
                 ||||||||||||||||||||||||||||||||||||||||||||||||||||||||||||
Sbjct  21134854  TTACTCTGGTTTGAATTTTTTGCATAGACATGAAAAAAGAAGAAGACCACACGTTGGCTC  21134913

Query  124       CGAATAAAACATGGCCGAGTCACCCTCCGAGTCAACCTCCGCGTTGCTTAGCACCACCAC  183
                 ||||||||||||||||||||||||||||||||||||||||||||||||||||||||||||
Sbjct  21134914  CGAATAAAACATGGCCGAGTCACCCTCCGAGTCAACCTCCGCGTTGCTTAGCACCACCAC  21134973

Query  184       ATCCACTAAGCCCGCTCAATCCGGGACTGTTTCTATTTCAAGCCCTCAAAGTCACCATGT  243
                 ||||||||||||||||||||||||||||||||||||||||||||||||||||||||||||
Sbjct  21134974  ATCCACTAAGCCCGCTCAATCCGGGACTGTTTCTATTTCAAGCCCTCAAAGTCACCATGT  21135033

Query  244       CGTCTTCCCTGAGATCCCAATCGAGATCATCAATGAAGAAGAAATAGTTATTCTCGATGC  303
                 ||||||||||||||||||||||||||||||||||||||||||||||||||||||||||||
Sbjct  21135034  CGTCTTCCCTGAGATCCCAATCGAGATCATCAATGAAGAAGAAATAGTTATTCTCGATGC  21135093

Query  304       AGCTCTTGCAGCCTCCCACTCCATCCTACCTTCCGTCATTCGTTCTGTTTCTCCGTCACA  363
                 ||||||||||||||||||||||||||||||||||||||||||||||||||||||||||||
Sbjct  21135094  AGCTCTTGCAGCCTCCCACTCCATCCTACCTTCCGTCATTCGTTCTGTTTCTCCGTCACA  21135153

Query  364       GATGATCGCCGGCGGAAGCCCAATAACTATTCGTTCAATTACTCTTTTCTCCAAGAGGAA  423
                 ||||||||||||||||||||||||||||||||||||||||||||||||||||||||||||
Sbjct  21135154  GATGATCGCCGGCGGAAGCCCAATAACTATTCGTTCAATTACTCTTTTCTCCAAGAGGAA  21135213

Query  424       ATTATCAGCTTGTTTATATATCGAAGAGTCTTATCTACATCGATTTAGAAGAAATCAGTC  483
                 ||||||||||||||||||||||||||||||||||||||||||||||||||||||||||||
Sbjct  21135214  ATTATCAGCTTGTTTATATATCGAAGAGTCTTATCTACATCGATTTAGAAGAAATCAGTC  21135273

Query  484       TTTAGGTGTCACAGATCTCACCGGTACTGTACGTTTGTTTTATATCACAGTCATATCATA  543
                 ||||||||||||||||||||||||||||||||||||||||||||||||||||||||||||
Sbjct  21135274  TTTAGGTGTCACAGATCTCACCGGTACTGTACGTTTGTTTTATATCACAGTCATATCATA  21135333

Query  544       GGAAGAAGAGATCGAATAGTAGCGACGACGTGGACAGCGGCTAATTGTGAGAGATGATTT  603
                 ||||||||||||||||||||||||||||||||||||||||||||||||||||||||||||
Sbjct  21135334  GGAAGAAGAGATCGAATAGTAGCGACGACGTGGACAGCGGCTAATTGTGAGAGATGATTT  21135393

Query  604       AGATTTAGTACAATTGTTAGGTTTAATATCTCTTTTTCTGGTGAGATTAATTTGTGGTCC  663
                 ||||||||||||||||||||||||||||||||||||||||||||||||||||||||||||
Sbjct  21135394  AGATTTAGTACAATTGTTAGGTTTAATATCTCTTTTTCTGGTGAGATTAATTTGTGGTCC  21135453

Query  664       ACTACTCATCCATTTCATTAATTTTCCATTTCATTGCTCATGTTATTTCATAATCTACAA  723
                 ||||||||||||||||||||||||||||||||||||||||||||||||||||||||||||
Sbjct  21135454  ACTACTCATCCATTTCATTAATTTTCCATTTCATTGCTCATGTTATTTCATAATCTACAA  21135513

Query  724       ACATATCTTTGGAATCTCACCATGATTGATAATTTCATTAATTCCAAAAATTAGAACTAA  783
                 ||||||||||||||||||||||||||||||||||||||||||||||||||||||||||||
```

Seqence-101821 (At5g60370)

```
GACGAGTCTAATTGGTATTGTGATACGTCTCATGGTGAAAAAAAAACCCCCCAGTACATTAAAAACGTC
CGCAATGTGTTATTAAGTTGTCTAAGCGTCAATTTGTTTACCCCACAATATATTGAGTATCCTCACCTG
GAAATGGATCACGGAGCCCAGTCTTTGTATCTATCAATATTGGTCCGCTATTAGAAGACTCTTCTGCAT
ACGCCTTTCTAATCTCGTCAATAATTCCCACAATCCATTGACCTCCAACAAATCCTAAGCTTTACAAGG
TAAAATGCACCATATTTAAAGTCTCATTTGATGTATGCTACAAAACATTATCTTGCTAGGAAAAGGTAC
AAACCACGAAAATGTTACAATAGAAGATCGAACCTAGAGGGGTAAAAGGAACTCACAGTACCTAATCAC
GAGTTCGTCCTTCAAACAAAAACTGATTAACACCAAAAATGGAATTCAACAGCTTCAATGCCCACTTAT
CTTCATTTGATTCAACTTTTACTCTCACTTTTTTAACTACCTACCCAATTATATAAACACAACAACAAG
TAGATTTCATCAAATTGTTTCACAAAACGATCACGTACTCTTAATAGTACCAATAAGCCCCTAAGATCC
ATCATAGTGAATAATACCTCTTCTTCAAGCTGCAAATGACGAGCCTGACCAACTTTTCATAGCTTTATT
GACTTTTCTCCTGCCAAACAAAGAACATTCTCCATTTTGTTTCTGCACTACCCACCGCGTTGCCCTAAT
AGTTGAGTGCCTTAATTTACAAGAC
```

Arabidopsis thaliana chromosome 5 sequence

Sequence ID: NC_003076.8 **Length:** 26975502 **Number of Matches:** 1

Range 1: 24283329 to 24283936 GenBank Graphics ▼ Next Match ▲ Previc

Score	Expect	Identities	Gaps	Strand
972 bits(526)	0.0	585/613(95%)	6/613(0%)	Plus/Minus

```
Query  131       CTCACCTGGAAATGGATCACGGAGCCCAGTCTTTGTATCTATCAATATTGGTCCGCTATT  190
                 |||| |||||| || ||||||||| | |||||||||||||||||||||||||||||||| 
Sbjct  24283936  CTCAGCTGGAAGTGTATCACGGAGTCGAGTCTTTGTATCTATCAATATTGGTCCGCTATC  24283877

Query  191       AGAAGACTCTTCTGCATACGCCTTTCTAATCTCGTCAATAATTCCCACAATCCATTGACC  250
                 ||||||||||||||||||||||||||||| ||||||||||||||||||||||||||||||
Sbjct  24283876  AGAAGACTCTTCTGCATACGCCTTTCTAAGCTCGTCAATAATTCCCACAATCCATTGACC  24283817

Query  251       TCCAACAAATCCTAAGCTTTACAAGGTAAAATGCACCATATTTAAAGTCTCATTTGATGT  310
                 ||||||||||||||||||||||||||||||||||| ||||||||||||||||||||||||
Sbjct  24283816  TCCAACAAATCCTAAGCTTTACAAGGTAAAATGCAACATATTTAAAGTCTCATTTGATGT  24283757

Query  311       ATGCTACAAAACATTATCTTGCTAGGAAAAGGTACAAACCACGAAAATGTTACAATAGAA  370
                 |||||||||||||||||||||||||||||||||| || |||||| |||||||||| ||||
Sbjct  24283756  ATGCTACAAAACATTATCTTGCTAGGAAAAGGTAAAAGCCACGAGAATGTTACAAAAGAA  24283697

Query  371       GATCGAACCTAGAGGGGTAAAAGGAACTCACAGTACCTAATCACGAGTTCGTCCTTCAAA  430
                 ||||||| ||||||||||| ||||||||||||||| | | ||||||||||||||||||||
Sbjct  24283696  GATCGAAACTAGAGGGGTAGAAGGAACTCACAGTAGCAATTCACGAGTTCGTCCTTCAAA  24283637

Query  431       CAAAAACTGATTAACACCAAAAATGGAATTCAACAGCTTCAATGCCCACTTATCTTCATT  490
                 ||| |||||||||||||||  |||||||||||||||||||||||||||||||||||||||
Sbjct  24283636  CAAGAACTGATTAACACCAGCAATGGAATTCAACAGCTTCAATGCCCACTTATCTTCATT  24283577

Query  491       TGATTCAACTTTTACTCTCACTTTTTTAACTACCTACCCAATTATATAAACACAACAACA  550
                 ||||||||||||||||||||||||||||||||||||||||||||||||||||||||||||
Sbjct  24283576  TGATTCAACTTTTACTCTCACTTTTTTAACTACCTACCCAATTATATAAACACAACAACA  24283517

Query  551       AGTAGATTTCATCAAATTGTTTCACAAAACGATCACGTACTCTTAATAGTACCAATAAGC  610
                 ||||||||||||||||||||| ||||||||||||| ||||||||||||||| ||||||||
Sbjct  24283516  AGTAGATTTCATCAAATTGTT-CACAAAACGATCAAGTACTCTTAATAGTAACAATAAGC  24283458

Query  611       CCCTAAGATCCATCATAGTGAATAATACCTCTTCTTCAAGCTGCAAATGACGAGCCTGAC  670
                 ||||||||||||||||||||||||||||||||||||||||||||||||||||||||||||
Sbjct  24283457  CCCTAAGATCCATCATAGTGAATAATACCTCTTCTTCAAGCTGCAAATGACGAGCCTGAC  24283398

Query  671       CAACTTTTCATAGCTTTATTGACTTTTCTCCTGCCAAA-CAAAGAACATTCTCCATTTTG  729
                 ||||||| |||||||||||||||||||||||||||||| ||||||||||||||||||| |
Sbjct  24283397  CAACTTT-CATAGCTTTATTGACTTTTCTCCTGCCAAAACAAAGAACATTCTCCATTT-G  24283340

Query  730       TTTCTGCACTACC  742
                 ||||| ||| |||
Sbjct  24283339  TTTCT-CAC-ACC  24283329
```
